# Multimodal machine learning reveals neurobiological signatures of binge-type eating disorders

**DOI:** 10.3389/fnins.2026.1803154

**Published:** 2026-04-13

**Authors:** Lena Rommerskirchen, Mandy Skunde, Martin Bendszus, Wolfgang Herzog, Hans-Christoph Friederich, Joe J. Simon

**Affiliations:** 1 Department of General Internal Medicine, Psychosomatics and Psychotherapy, Centre for Psychosocial Medicine, University Hospital Heidelberg, Heidelberg, Germany; 2 Institute of Pathology, University Hospital Heidelberg, Heidelberg, Germany; 3 Department of Neuroradiology, University Hospital Heidelberg, Heidelberg, Germany; 4 DZPG (German Centre for Mental Health – Partner Site Heidelberg/Mannheim/Ulm), Germany

**Keywords:** binge eating disorder (BED), bulimia nervosa (BN), fMRI, functional connectivity, machine learning (ML)

## Abstract

**Introduction:**

Binge-type eating disorders, including bulimia nervosa (BN) and binge eating disorder (BED), are associated with both shared and disorder-specific neurobiological mechanisms across brain, behavior, and physiology. A clearer distinction between shared mechanisms and disorder-specific alterations may advance our understanding of binge-type eating pathology.

**Methods:**

We applied a comprehensive multimodal machine learning framework to 110 participants (BN, BED, and age & weight matched controls), integrating task-based fMRI, intrinsic connectivity, voxel-based morphometry, neuropsychological assessments, and peripheral blood biomarkers. Both unimodal and multimodal machine learning models were trained to classify groups and to predict individual variation in symptom expression.

**Results:**

Functional brain connectivity achieved the highest accuracy for diagnostic classification and symptom prediction (with a mean balanced classification accuracy (bACC) of 68.7%), whereas task-based fMRI with disorder-specific food stimuli and peripheral blood biomarkers best distinguished BN from BED (mean bACC of 87%). Multimodal models did not generally outperform the best unimodal approaches, except from modest gains in a limited set of regression targets.

**Conclusions:**

These findings suggest that functional brain connectivity carries robust predictive information for transdiagnostic classification, whereas task-evoked activation patterns and peripheral biomarkers show stronger predictive utility for distinguishing BN from BED. Whether these modality-specific patterns reflect underlying neurobiological mechanisms remains to be established in future hypothesis-driven work. Identifying which modalities best represent shared vulnerability vs. symptom-type-dependent variation may help to provide a foundation for a more mechanistic understanding of these disorders.

## Introduction

Eating disorders (EDs) are a complex group of psychosomatic conditions marked by dysfunctional eating behaviors, a preoccupation with weight and shape, metabolic and endocrine disruptions, and body mass indices (BMIs) ranging from underweight to obesity. Individuals with EDs show substantial variability in both clinical presentation and underlying neurobiology, with symptom dimensions often transcending diagnostic boundaries (Carr and Grilo, 2020; Arend et al., 2023; Tiego et al., 2023; Christensen Pacella et al., 2025). Among these, bulimia nervosa (BN) and binge eating disorder (BED) share recurring episodes of uncontrolled binge-eating accompanied by distress, guilt, and shame, yet differ in their use of weight control strategies. BN is characterized by compensatory purging behaviors such as self-induced vomiting, excessive exercise or the misuse of laxatives and diuretics to prevent weight gain, whereas individuals with BED do not engage in purging and are typically overweight (American Psychiatric, 2013).

Binge eating has been consistently associated with widespread disruptions in neural systems related to reward processing, affect regulation and cognitive control (Smith et al., 2018; Wonderlich et al., 2021), although its exact pathophysiology remains unclear. Compared to healthy controls, binge-eating has been linked to reduced inhibitory control (Skunde et al., 2016; Giel et al., 2022; see Van den Eynde et al., 2011, for mixed results) and attentional and cognitive bias toward food-related cues (Schag et al., 2013; Stojek et al., 2018; Li et al., 2022; Leehr et al., 2023) indicating a heightened subjective valuation of these stimuli. In line with this, frontostriatal connectivity and striatal dopaminergic activity are also attenuated (Haynos et al., 2021; Yu et al., 2022; Wang et al., 2023). These findings have been integrated into multistage models of food-related decision-making, in which the interactions between valuation, action selection, learning, and regulatory control determine the trajectory of disordered eating habits (Colton et al., 2023; Schaefer et al., 2023). For instance, increased hedonic valuation of food can intensify attentional bias toward food-related cues while weakened inhibitory control may diminish the ability to regulate such impulses. This can foster cycles of craving and loss-of-control eating, particularly when negative affect or stress limit top-down regulation (Leehr et al., 2023) or when negative consequences are not effectively encoded in reinforcement learning processes, thereby contributing to the habitual and persistent nature of binge-eating (Voon et al., 2015). However, there is limited evidence that these mechanisms differ between disorders. Specifically, while both disorders show weakened coupling within frontostriatal circuits involved in self-regulatory control, BN tends to exhibit stronger connectivity of medial prefrontal and anterior cingulate regions (Stopyra et al., 2019), reflecting increased engagement of control and conflict-monitoring systems (Ridderinkhof et al., 2004), whereas BED is characterized by stronger connectivity within striatal and orbitofrontal regions linked to reward learning and hedonic valuation (Ahn et al., 2022; Leenaerts et al., 2022). Together, these findings suggest that BED may represent a more reward-driven loss of control, whereas BN involves heightened tension between reward drive and compensatory control efforts, leading to distinct behavioral expressions of impulsivity (Giel et al., 2017; Ahn et al., 2022).

Recently, machine learning (ML) approaches have gained traction as powerful tools to model these distributed and interacting mechanisms (Rashid and Calhoun, 2020). Unlike traditional univariate analyses, ML can integrate diverse data types and capture complex, non-linear relationships across modalities without relying on strong theoretical assumptions (Koppe et al., 2021; Chen et al., 2022). This is particularly relevant for psychiatric disorders, where cognitive and affective domains are inherently nested and involve subprocesses with distinct temporal and physiological signatures (Insel et al., 2010). A few studies have begun to apply ML approaches to binge eating, including work by Levinson and colleagues (Levinson et al., 2023), who used ML classifiers on self-report variables to predict clinical behaviors such as binging or purging with moderate to high accuracy. Similarly, behavioral measures have been shown to distinguish recurrent from non-recurrent binge-eating (Linardon et al., 2020), and physiological and clinical features have been combined to differentiate individuals with BED from weight-matched controls (Rania et al., 2025). In contrast, other studies reported that ML models provided little improvement over traditional regression approaches in predicting treatment outcomes such as binge abstinence and weight loss (Forrest et al., 2023). While these findings demonstrate the potential of ML for identifying clinically relevant features in binge-eating, most studies have been limited to single modalities and narrowly defined prediction targets. Consequently, it remains unclear how distinct data types capture shared vs. disorder-specific aspects of binge-type eating pathology.

Therefore, this study aimed to systematically examine how different data modalities represent categorical and dimensional aspects of binge-type EDs within a single, well-characterized sample. It is the first study to apply a multimodal ML framework in a cohort of 110 participants, including individuals with BN, BED, and healthy controls. In line with the Research Domain Criteria (RDoC; Insel et al., 2010) framework, which emphasizes mapping relationships between functional domains and multiple levels of analysis to better understand transdiagnostic and diagnosis-specific mechanisms, we sought to describe systematic patterns in how distinct modalities capture variation across diagnostic groups and symptom dimensions. To this end, we used a dataset including structural and functional neuroimaging, neuropsychological task-based measures, peripheral blood biomarkers, and clinical questionnaires, and developed multiple ML models differing in both the type of data used and the clinical features predicted.

By analyzing prediction targets such as binge eating within BN, within BED, and in pooled samples, we assessed whether common neurobiological mechanisms underlie this transdiagnostic construct. As a secondary aim, we tested whether integrating modalities improves predictive accuracy, under the assumption that different data types capture complementary and interacting aspects of shared neurobiological processes (Rashid and Calhoun, 2020; Iceta et al., 2021; Boehm et al., 2022; Colombo et al., 2022).

## Materials and methods

### Participants

This study reanalyzed fMRI data from a single cohort previously investigated by our group in studies investigating food- and monetary-related reward processing (Simon et al., 2015, 2016), general- and food-specific inhibition (Skunde et al., 2016), as well as functional and seed-based connectivity (Stopyra et al., 2019) in patients with BED and BN. Additionally, unpublished peripheral blood biomarkers and neuropsychological measures of inhibition and working memory were included in the analysis. One hundred nineteen individuals participated in the study originally. We pre-excluded 9 participants because of incomplete demographic information. In total, data from 110 right-handed participants over the age of 18 were analyzed: 26 patients meeting ICD-10 criteria for BED, 29 patients meeting ICD-10 criteria for BN, and 55 healthy control participants. The control group consisted of two subgroups, each matched to either the BED (owHC; overweight healthy controls) or BN (nwHC; normal weight healthy controls) group on age, sex, BMI, and education. Four subjects in the BED group and five subjects in the owHC group were male. Demographic and clinical characteristics of participants are given in Supplementary Table 1. Exclusion criteria included claustrophobia, metallic implants, and lifetime diagnoses of bipolar disorder, borderline personality disorder, psychosis, alcohol or drug abuse. Five participants in the BED group and seven participants in the BN group were receiving antidepressant medication. Patients were recruited from our wards and outpatient clinic, while healthy controls were recruited via advertisements. The study was approved by the local ethics committee of the Medical School of the University of Heidelberg. All participants provided written and oral informed consent.

### ML-framework

**Figure 1:**
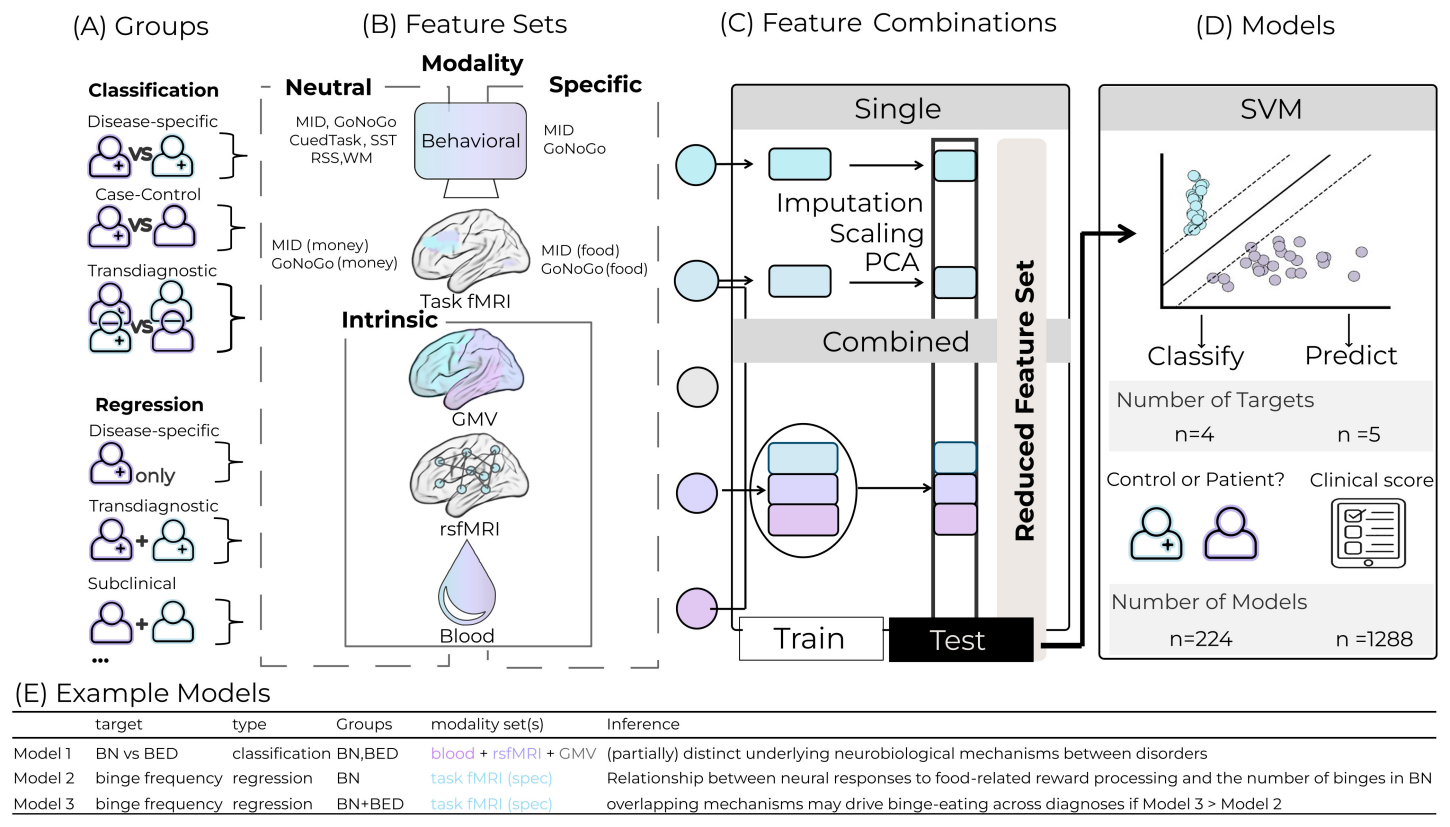
Overview of the machine learning framework. ML models were constructed using unique combinations of modality feature sets from various participants groups for each target. To account for patient variability, ML models were trained on different combinations of subject groups **(A)**. Discrimination between BN and BED is successful when the model features capture specific aspects of the diagnostic categories. In contrast, case-control classifications (e.g., BN vs. nwHC) reflect features related to patient status without making further assumptions about their specificity to the disorder. Discrepancies between pooled patient and control groups are indicative of transdiagnostic features. We used Data were obtained from multiple modalities **(B)**, including behavioral tests, brain imaging (structural MRI, resting-state and task-based fMRI), and peripheral blood biomarkers. Task-based data were further categorized based on stimuli type (neutral or food-related/specific) and neutral, specific and intrinsic sets for non-task data. Models were built using either single modalities or combinations of modalities within and across sets, except for combinations of neutral and specific sets, which were excluded to reduce the overall number of feature sets. **(C)** After data transformation and feature reduction, models were trained and tested using support vector machines (SVMs). **(D)** In total, four diagnostic classification targets and five symptom prediction targets were examined across 189 unimodal and 1,323 multimodal models. For each model, a corresponding dummy model was employed to assess the significance of above-chance performance. BN, Bulimia nervosa; BED, Binge-eating disorder; HC, Healthy controls; sMRI, Structural magnetic resonance imaging; rsfMRI, Resting-state functional magnetic resonance imaging; task fMRI, Task-based functional magnetic resonance imaging; MID, Monetary Incentive Delay task; Go/NoGo, Response-inhibition task; SVM, Support vector machine. Examples of modality features sets are given in **(E)**.

To evaluate which types and combination of features best capture clinically relevant differences between groups and predict individual symptom severity, we created a comprehensive ML framework consisting of 1,512 independent ML models (see Figure 1 and Table 1 for included features and detailed model explanations).

**Table 1:** Columns list the modality, specific feature set, stimulus type (neutral = monetary/unspecific cues; specific = disorder-relevant food cues; intrinsic = no external stimulus), the number of features per set, and the number of subjects available for that modality.

Modality	Stimuli	Features	N features	N subjects
Behavioral	Neutral	Cued task	4	110
		GNG	4	
		MID	3	
		RSS	4	
		Stop signal	3	
		Intelligence	1	
		WM	2	
	Specific	MID	3	107
		GNG	4	
Peripheral blood biomarkers	Intrinsic		6	107
fMRI	Neutral	GNG	276	108
		MID	1,370	
	Specific	GNG	276	
		MID	1,380	
	Intrinsic	RS	13,695	
MRI	Intrinsic	GMV	166	109

Exclusions were made independently for each feature set, therefore sample sizes vary slightly across modalities. GNG, Go/NoGo; MID, Monetary Incentive Delay; RSS, Reward Sensitivity Scale; GMV, Gray-matter volume; fMRI, Functional magnetic resonance imaging; MRI, Magnetic resonance imaging; RS, resting-state; N features, number of features; N subjects, number of subjects.

### Targets

To assess disease-specific as well as transdiagnostic factors, four diagnostic group contrasts were defined: (i) BN vs. nwHC, (ii) BED vs. owHC (case–control), (iii) BED vs. BN (disease-specific case–case), and (iv) Patients vs. Controls (transdiagnostic; BN+BED pooled vs. both control groups pooled). Composite scores from questionnaires and clinical data served as severity indices for four symptom domains. Because disease severity is linked to disease duration (Austin et al., 2021; Miskovic-Wheatley et al., 2023; Robinson et al., 2024), we additionally computed a fifth factor with variables related to lifetime weight fluctuations. The symptom domains included were defined as follows: *Disease-unspecific:* general depressive symptoms as measures by the BDI (Hautzinger et al., 2006).

*Eating-unspecific:* general eating behaviors measured with the DEBQ (Grunert, 1989) and FCQ (Nijs et al., 2007) subscales, combined using equal weights.

*Eating-specific pathology:* Eating disorder–specific psychopathology indexed by the EDEQ (Hilbert et al., 2004) total score.

*Binge-specific:* self-reported frequency of binge eating episodes per week.

*Weight fluctuations and weight monitoring behavior:* Reflects the quantity of weight fluctuations (e.g., ±5, ±10 kg, etc.) across the lifespan and the frequency of self-weighings, each contributing 50% to the composite score.

We accounted for within and between group heterogeneity by training models based on data from different groups of participants. For instance, the frequency of binge episodes can be predicted either within BN and BED separately or by combining both groups of patients. The composition of the sample therefore reflects different levels of variability in the input features. If BN and BED share similar underlying mechanisms, combining groups should yield similar or better predictive accuracy due to the larger sample size. If, however, the mechanisms differ, combined models may perform worse because adding heterogeneous cases introduces additional error variance.

### Feature set construction

Features for this analysis were derived from behavioral, neuroimaging, and physiological measures of eating behavior and reward processing (Table 1). A detailed description of feature extraction and univariate analyses is provided in Supplementary material C, D. Briefly, behavioral data were obtained from neuropsychological tests assessing inhibitory control (SST, Stop Signal Task, designed to assess motor inhibition; Verbruggen et al., 2008), cognitive control related to response inhibition (RSS, Response Set Shifting task; Monsell, 2003), cognitive flexibility (CSS, Cued Set Switching; Meiran, 1996), short-term and working memory (WMS-DS, Wechsler Memory Scale Digit Span subtest; Petermann and Lepach, 2012), and premorbid verbal intelligence (MWT-B, Multiple-Choice Vocabulary Intelligence Test; Lehrl et al., 2005). Task fMRI-features comprised regional activation estimates from the Monetary Incentive Delay task (MID) and Go/NoGo task under *neutral* (monetary) and *disorder-specific* (food-related) conditions (Simon et al., 2015, 2016). Brain functional connectivity was derived from resting-state fMRI (rsfMRI) data using a parcel-wise functional connectivity approach following Weis et al. (2020) to reduce the dimensionality of the feature space. Predictive modeling was applied separately to each brain parcel and clinical target, and the 10 parcels showing the strongest predictive performance for each target were retained. Structural MRI (sMRI) features reflected mean gray-matter volume (GMV) extracted from T1-weighted anatomical images. Peripheral blood parameters included metabolic and endocrine markers consistently linked to appetite and weight regulation (Eberle et al., 1991; Suh et al., 2013; Chen et al., 2019; Maher et al., 2019; Wyatt et al., 2021; Vigil et al., 2022), specifically glucose, γ-glutamyltransferase (GGT), triglycerides, total cholesterol, progesterone, and estradiol (E2). All neuroimaging features were based on parcellation according to the AAL3 atlas (Rolls et al., 2020), except for task-fMRI, where thalamic nuclei were replaced by the AAL2 parcellation of the thalamus.

To examine the added value of combining different data types, features were grouped by source: neuropsychological behavioral tests, task-fMRI, brain structure, rsfMRI, and blood markers. Behavioral and task-fMRI features were further divided by stimulus type (neutral or food-related). Modalities without external stimulation (structural MRI, rsfMRI, blood) were classified as intrinsic and treated as relevant to both categories. Prediction models were trained using single feature sets and selected multimodal combinations, with an additional model including all features. This hierarchical setup minimized redundancy and emphasized complementary information across modalities.

### Model evaluation

Single modality and multimodal models for all prespecified group-feature-target combinations were implemented in Python using scikit-learn (v1.5.2) and julearn (https://juaml.github.io/julearn/ v0.3.4). Support Vector Machines (SVM) were employed for classification and Support Vector Regression (SVR) for continuous outcomes. These were chosen for their robustness with moderate sample sizes and mixed feature spaces (Awad and Khanna, 2015; Guido et al., 2024). Due to sample size considerations and to avoid overfitting risks associated with extensive hyperparameter optimization in relatively small neuroimaging samples (Varoquaux et al., 2017), no tuning loop was implemented. Instead, scikit-learn's default hyperparameter settings were used for all models, which have been shown to be generally robust across a wide range of datasets (Poldrack et al., 2020).

Model evaluation was conducted using a five-fold repeated, group-stratified three-fold cross-validation (CV) procedure (15 test folds) to estimate generalizability. This approach was chosen over leave-one-subject-out CV to better accommodate heterogeneity in patient groups (Varoquaux et al., 2017; Poldrack et al., 2020). All preprocessing steps were performed within CV folds to prevent data leakage and train/test splits were held constant across models for comparability. Missing values in feature vectors were median-imputed. BMI was included as a confounding factor for contrasts in which groups differed substantially in body weight (e.g., BED vs. BN and regression analyses), whereas BMI was not included in case–control contrasts where control groups were matched for BMI (e.g., BED vs. owHC and BN vs. nwHC) to avoid over-adjustment. The data were subsequently scaled with scikit-learn's *RobustScaler* to account for variability in the data. Dimensionality reduction was performed using principal component analysis (PCA), with the number of components bounded by fold sample size and feature dimensionality, targeting approximately 80% retained variance. For rsfMRI-data, a hypergeometric enrichment analysis (Bunnik et al., 2016) was performed to test whether the 10 most predictive parcels clustered within specific functional systems and differed by prediction target (further details are given in the Supplementary material).

For multimodal setups, a late fusion strategy was adopted by performing scaling and PCA separately for each modality set. The resulting component scores (with a common component count across sets determined by the minimum feasible number) were concatenated before model fitting. Performance was summarized using balanced accuracy (bACC) for classification and *R*^2^ for regression. To determine whether each model performed significantly above chance, a dummy model was trained for every model to determine baseline prediction levels. This model predicts class labels based on the class distribution of the stratified training sets. Between-model comparisons were carried out using the model-corrected paired *t*-test (Nadeau and Bengio, 2003) as implemented in julearn, which adjusts variance estimates for dependence introduced by overlapping training/test splits. Statistical significance was set to α = 0.05. First, fold-wise *t*-tests against the dummy baseline were conducted for all single and multimodal models per target. Second, to evaluate whether combining feature sets improved model performance, the best single-modality model per target was compared with each significant multimodal candidate using fold-wise tests (Welch's *t* for unequal variances; paired *t* when fold alignment allowed) if the candidate showed a higher mean bACC or *R*^2^.

## Results

### Unimodal classification

**Figure 2:**
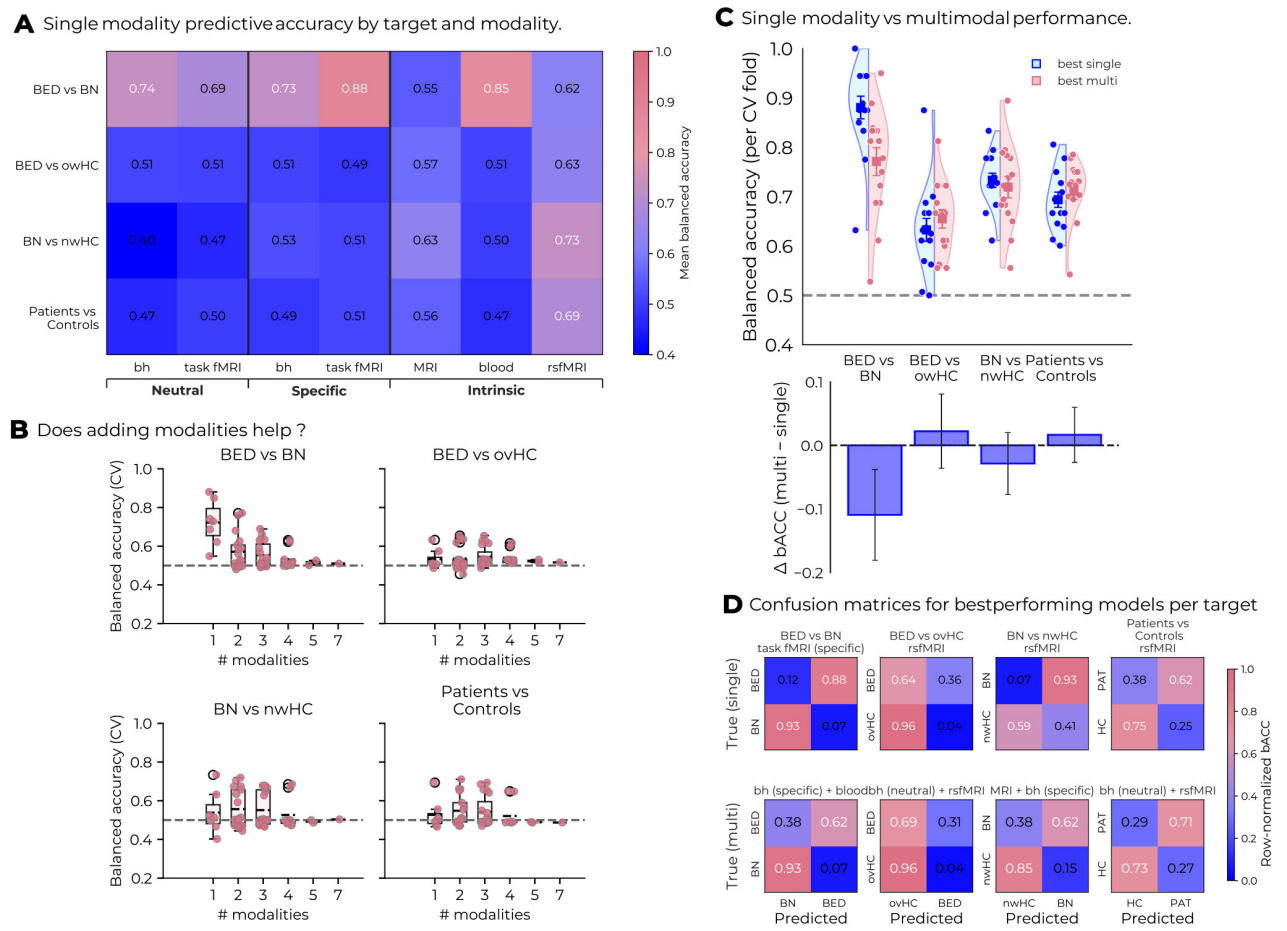
Summary of classification performances across modalities and targets. **(A)** Heatmap of mean cross-validated balanced accuracy (bACC) for single-modality models, organized by stimulus class (neutral, specific, intrinsic). Task-fMRI with food stimuli and peripheral blood biomarkers differentiate between BED and BN, whereas rsfMRI separates patients from controls. **(B)** Relationship between performance and the number of modalities included. Accuracy does not increase monotonically with added modalities; gains occur only for select combinations. The dashed line marks chance (bACC = 0.5). **(C)** Comparison of the best single (blue) and best multimodal (pink) models for each target. Split violin plots show per-fold bACC. Squares denote mean ± SEM. The bar plot below shows ΔbACC (multi minus single), highlighting that fusion yields small or no average improvements for most targets. **(D)** Row-normalized confusion matrices for each target: top shows the best single-modality model, bottom shows the best multimodal model. Values indicate the proportion of true-class samples assigned to each predicted class. bACC, balanced accuracy; rsfMRI, resting-state functional MRI; task-fMRI, task-based functional MRI; MRI, structural MRI (GMV); bh, behavioral; BED, binge-eating disorder; BN, bulimia nervosa; owHC, overweight/obese healthy controls; nwHC, normal-weight healthy controls; HC, healthy controls; PAT, patients.

Unimodal classification models that performed significantly above chance are presented in Supplementary Table 8 and illustrated in Figure 2. Overall, significant above-chance performance was observed in 20.54% (47 out of 224) of unimodal classification models. As shown in Figure 2A, the best-performing modality was rsfMRI, which successfully distinguished between patient-control contrasts: bACC was 0.633 for BED vs. ovHC and 0.733 for BN vs. nwHCs (both above chance; *p* = 0.036 and *p* < 0.001, respectively). For all patients vs. controls combined, rsfMRI reached 0.694 (*p* < 0.001). For the disease-specific comparisons (BN vs. BED), models with peripheral blood biomarkers and task-based fMRI with food stimuli significantly performed above chance (bACC ≈ 0.88, both *p* < 0.001). Notably, rsfMRI did not distinguish BN from BED (*ps* > 0.05). As shown in the confusion matrices (Figure 2D), in case-control comparisons, controls were generally classified more accurately than patients. In the disease-specific BN vs. BED contrast, the best single-modality model yielded high accuracy with symmetric error distribution. Pooled patient-control classification remained balanced across classes.

### Multimodal classification

For each classification target, we compared all multimodal combinations that performed above chance with the corresponding best single-modality reference model. The results are summarized in Table 2 and illustrated in Figure 2. Although several multimodal models achieved above-chance performance (see Supplementary Table 9), none showed a statistically significant improvement over the single-modality baselines. As shown in Figure 2B, overall accuracy did not increase monotonically with the number of combined modalities, suggesting that adding additional data sources may introduce redundancy rather than complementary information. Consistent with this, Figure 2C shows that fusion models (pink) rarely outperformed the best single-modality counterparts (blue), with average gains near zero across contrasts. Small but non-significant improvements were observed for a subset of combinations that included rsfMRI features. The largest gains were found for the BED vs. owHC (ΔbACC = +0.022) and patients vs. controls contrast (ΔbACC = +0.017), both combining behavioral and rsfMRI features (Table 2).

**Table 2:** Values indicate mean balanced accuracy (bACC) across cross-validation folds.

Target	Modalities	Best single model bACC	bACC	*p* Welch	*p* paired-*t*
BED vs. owHC	bh (neu)+rsfMRI	0.633	0.655	0.459	0.514
	GMV+bh (spec)+rsfMRI		0.654	0.527	0.552
	bh (spec)+rsfMRI		0.639	0.825	0.832
	GMV+rsfMRI		0.636	0.9	0.892
Patients vs. controls	bh (neu)+rsfMRI	0.694	0.711	0.457	0.544
	GMV+bh (neu)+rsfMRI		0.694	0.997	0.998

Best single model bACC refers to the highest-performing unimodal reference model for the same target (see Supplementary Table 8). None of the multimodal combinations yielded statistically significant improvements (all *p* > 0.45). bACC, balanced accuracy; bh, behavioral; neu, neutral task condition; spec, disorder-specific (food) task condition; rsfMRI, resting-state functional MRI; GMV, gray-matter volume; HC, healthy controls; owHC, overweight/obese healthy controls.

### Unimodal regression

**Figure 3:**
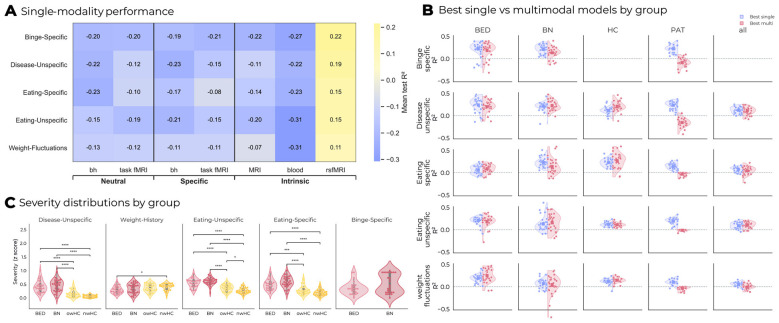
Regression results for symptom severity targets. **(A)** Heatmap of mean test *R*^2^ from single modality models for each target by modality; modalities are arranged by stimulus context (neutral, specific) and non-task measures (intrinsic). **(B)** For each target (rows) and cohort (columns: BED, BN, HC, PAT), split-violin plots compare the best single-modality model (left) with the best multimodal model (right). Points show per-fold scores and squares denote mean ± SEM. The gray dashed line marks *R*^2^ = 0. **(C)** Distributions of observed severity scores by diagnostic group (violin and swarm plots). Black brackets indicate significant Tukey HSD pairwise differences. BED, binge eating disorder; BN, bulimia nervosa; HC, healthy controls; PAT, patients (all eating-disorder patients); *R*^2^, coefficient of determination; SEM, standard error of the mean; bh, behavioral measures; task fMRI, task-based functional MRI; rsfMRI, resting-state functional MRI; MRI, structural MRI; ANOVA, analysis of variance; Tukey HSD, Tukey's honestly significant difference. ^*^p < 0.05; ^**^p < 0.01; ^***^p < 0.001; ^****^p < 0.0001.

As shown in Figure 3 and Table 3, only rsfMRI yielded consistent above-chance prediction across symptom severity targets. In total, 6.21% of unimodal regressions were significant. Mean test *R*^2^ values for rsfMRI were 0.23 for *Eating-specific severity*, 0.21 for *Eating-unspecific*, and 0.19 for Weight-fluctuations and monitoring, indicating that rsfMRI features alone explained roughly 19–23% of the variance across outcomes. Group-wise prediction accuracy showed heterogeneous patterns: for some symptom targets, pooled patient samples achieved higher *R*^2^ values, whereas for others, separate BN or BED models performed better, indicating that predictive signal strength varied with both group and target (see Supplementary Figure 2). In contrast, behavioral, task-based, and structural modalities showed near-zero predictive accuracy (see Figure 3A). Univariate analyses of regression targets are shown in Figure 3C. Significant group differences were observed for most targets, indicating that regression targets captured clinically meaningful variation. As expected, BN and BED patients showed elevated symptom levels relative to controls across all domains. Interestingly, weight-fluctuations and monitoring behavior differed significantly only between BED and normal-weight controls, but not between BN and their matched controls. This pattern suggests that this outcome primarily reflects long-term weight variability associated with higher body mass, rather than binge-type symptom expression.

**Table 3:** Only models with statistically significant prediction are shown; all other regression models were not significant.

Target	Group	Confounds	Modality	bACC	*t*	*p*
Binge-specific	PAT	BMI	rsfMRI	0.221	3.691	0.000
	BED	BMI	rsfMRI	0.218	2.631	0.007
	BN	BMI	rsfMRI	0.209	4.125	0.000
Disease-unspecific	PAT	None	rsfMRI	0.233	4.240	0.000
	HC	None	rsfMRI	0.119	2.269	0.015
	All	None	rsfMRI	0.123	2.508	0.009
	BED	None	rsfMRI	0.239	2.932	0.003
	BN	None	rsfMRI	0.211	3.228	0.002
Eating-specific	PAT	BMI	rsfMRI	0.141	3.924	0.000
	HC	BMI	rsfMRI	0.227	4.324	0.000
	All	BMI	rsfMRI	0.075	1.991	0.028
	BN	BMI	rsfMRI	0.230	3.995	0.000
Eating-unspecific	PAT	BMI	rsfMRI	0.194	5.251	0.000
	HC	BMI	rsfMRI	0.119	3.646	0.001
	All	BMI	rsfMRI	0.093	2.011	0.027
	BED	BMI	rsfMRI	0.208	3.203	0.002
Weight-fluctuations and monitoring	PAT	BMI	rsfMRI	0.100	2.644	0.007
	HC	BMI	rsfMRI	0.121	2.927	0.003
	All	BMI	rsfMRI	0.055	1.849	0.037
	BED	BMI	rsfMRI	0.189	3.254	0.001
	BN	BMI	rsfMRI	0.068	1.791	0.042

“Confound” indicates whether BMI-related variance was removed prior to modeling (“BMI”) or not (“None”). BN, Bulimia nervosa; BED, Binge-eating disorder; HC, Healthy controls; PAT, Patients (BN+BED combined); all, Patients+healthy controls; rsfMRI, Resting-state functional MRI; GMV, Gray-matter volume (structural MRI); BMI, Body mass index.

### Multimodal regression

Single-modality rsfMRI models served as baselines for evaluating multimodal combinations. Results are listed in Supplementary Tables 10–15. While adding additional modalities did not improve predictive accuracy for Eating-specific or Eating-unspecific severity (Δ*R*^2^ ≈ 0.00–0.04; all *p* > 0.30, Welch and paired *t*-tests, see Figure 3B), several multimodal combinations yielded significant gains for weight-related and disease-unspecific outcomes. In patients with BED, combining resting-state functional connectivity with peripheral blood biomarkers (Δ*R*^2^ ≈ +0.06; paired-*t p* = 0.038) or with both blood and food-specific behavioral features (Δ*R*^2^ ≈ +0.06; paired-*t p* = 0.043) significantly improved prediction of lifetime weight fluctuations and monitoring behavior (mean *R*^2^ ≈ 0.25). In healthy controls, smaller but consistent improvements were observed for the same outcome when rsfMRI was combined with structural and behavioral measures (Δ*R*^2^ ≈ +0.02–0.03; paired-*t p* ≤ 0.04; mean *R*^2^ ≈ 0.14). Additionally, multimodal integration enhanced the prediction of disease-unspecific (depressive) symptom severity in healthy controls when rsfMRI was combined with behavioral and structural MRI features (Δ*R*^2^ ≈ +0.09; paired-*t p* < 0.001). No other combinations yielded significant improvements over the best single-modality reference models (Δ*R*^2^ ≤ ~0.04; *p* ≥ 0.25).

### Network-level enrichment of predictive brain functional connectivity parcels

To evaluate network-level patterns among the top predictive rsfMRI parcels, we performed a network enrichment analysis. Results showed default-mode network (DMN) enrichment for the classification of patients vs. controls, as well as visual–frontal–cerebellar enrichment for the prediction of disease-unspecific symptom severity, and temporal/limbic–striatal enrichment for the prediction of BN-specific severity. Detailed results are provided in Supplementary Results E, Supplementary Tables 2–7, and Supplementary Figure 1.

## Discussion

Binge-type EDs are characterized by widespread alterations in neural, behavioral, and physiological systems, yet an integrative framework linking these changes to disease mechanisms remains elusive. Building on recent advances in predictive modeling aimed at capturing clinical heterogeneity, the present study is the first to integrate multiple data modalities to examine how neural, behavioral, and peripheral measures relate to diagnostic classification and symptom expression in binge-type EDs. We applied a unified predictive modeling framework in which different modalities were analyzed both separately and jointly, including resting-state functional connectivity, gray matter volume (GMV), task-based activation during reward-, food-, and inhibition-related paradigms, peripheral blood biomarkers, and neurocognitive performance indices. We found that functional brain connectivity provided the strongest information for distinguishing patients from healthy controls, whereas task-based fMRI and peripheral blood biomarkers best differentiated BN from BED. Although multimodal integration did not improve categorical classification beyond the best unimodal models, it enhanced the prediction of several symptom related outcomes, particularly when combining functional connectivity with peripheral or behavioral measures. These findings suggest that predictive information in binge type eating disorders is largely modality specific, yet selective symptom dimensions can benefit from cross modal integration.

Across all analyses, functional brain connectivity was the strongest predictor across both classification and regression outcomes. While functional connectivity is often analyzed at the network level, referring to coordinated activity among large-scale networks such as the default-mode and salience networks (Menon, 2011), the present study focused on parcel-wise connectivity profiles, defined as the pattern of connections between individual regions and the rest of the connectome (Weis et al., 2020). Exploratory network-enrichment analyses indicated predominant involvement of the default-mode network (DMN) and related large-scale systems implicated in self-referential processing and emotion regulation (Davey et al., 2016; Oliva et al., 2020; Ahn et al., 2022). The DMN has been consistently linked to transdiagnostic alterations across eating-disorder phenotypes (Steward et al., 2018; Leenaerts et al., 2022), raising the possibility that disruptions within these networks may reflect a transdiagnostic neural vulnerability relevant to binge-type pathology.

Furthermore, we found that in contrast to functional brain connectivity, food-related task-based fMRI and peripheral blood biomarkers were able to distinguish BN from BED. This aligns with prior work showing that differences within binge-type ED arise through more localized coupling alterations, particularly within frontostriatal and anterior cingulate circuits related to reward and inhibitory control (Schienle et al., 2009; Schafer et al., 2010; Leenaerts et al., 2022). Therefore, our results suggest that task-based fMRI, which directly engages these circuits, may be better suited to detect diagnostic specificity. Given that this was only observed in the food-related versions of the reward and inhibition tasks used in this study, a possible explanation is that food cues exerted a stronger and more disorder relevant salience than the monetary stimuli in these paradigms. In fact, food images reliably elicit heightened motivational relevance and stronger affective engagement than neutral or monetary conditions in patients with ED (Berridge and Robinson, 2016; Bodell and Racine, 2023). Furthermore, evidence from other mental disorders shows that when stimuli closely relate to the core symptoms of a disorder, they tend to elicit broad salience and arousal responses that can overshadow disorder-specific task effects (Feldker et al., 2017; MacNamara et al., 2017). Therefore, in the present study, food cues may have recruited broad salience and arousal systems that are largely shared across all groups, which could explain the limited patient vs. control separation (Sescousse et al., 2013; Banica et al., 2022; Lawhead et al., 2025). However, BN and BED differ in their disorder specific responses to food cues within reward and inhibitory control circuits, allowing BN vs. BED distinctions to emerge despite the absence of clear patient vs. control differences.

The difference between task-based and brain-connectivity findings highlights the importance of the type of variance captured by each modality. The predictive models used in this study rely on between-subject differences, yet symptom expression in EDs often fluctuates substantially within individuals over time (Levinson et al., 2023). This instability is evident in the high rates of diagnostic crossover across ED subtypes (Tozzi et al., 2005; Eddy et al., 2008). Temporal alignment between modalities may also play a role. Task-based activations reflect transient cognitive–affective states elicited during scanning, whereas symptom severity scores such as binge frequency summarize behavior across longer periods. This temporal mismatch may weaken observed brain–symptom associations in task-based models. In contrast, brain functional connectivity, which reflects more stable, trait-like aspects of brain organization (Laumann et al., 2017; Pannunzi et al., 2017; Sbaihat et al., 2022), may be better suited for predicting aggregated symptom severity. Together, these findings suggest that large-scale network connectivity, particularly within the DMN, may characterize general disease status, whereas regionally specific connectivity and task-evoked activations differentiate diagnostic subtypes.

A further aim was to test whether integrating multiple modalities enhances predictive accuracy beyond single modality models. Although multimodal fusion is theorized to improve performance by combining complementary neural, behavioral, and peripheral information (Rashid and Calhoun, 2020; Iceta et al., 2021; Boehm et al., 2022; Colombo et al., 2022), gains were modest and restricted to regression targets related to weight and affective symptom load. Benefits emerged mainly when peripheral blood biomarkers were combined with neuroimaging measures. In BED, this likely reflects heightened physiological strain and a stronger coupling between peripheral metabolic and neural processes involved in weight regulation (Montani et al., 2006; Dulloo and Montani, 2015), consistent with evidence linking repeated weight cycling to systemic inflammation and metabolic dysregulation (Ilyas et al., 2019; Iceta et al., 2021; Rania et al., 2025). In healthy controls, improvements may instead arise from greater temporal stability and homogeneity of neural signal, which can strengthen the mapping between trait like connectivity patterns and relatively stable target variables such as affective state or weight monitoring. By contrast, no multimodal benefit was observed in BN, possibly because unimodal functional connectivity models performed near chance. When baseline prediction is weak, adding additional non-significant modalities primarily increases dimensionality without improving the feature to sample size ratio, thereby reducing predictive stability (Colombo et al., 2022; Mattoni et al., 2025). Overall, our findings indicate that single modalities often capture most of the discriminative variance, so additional data types provide limited added value in moderate sized or heterogeneous samples where redundancy and individual variability can obscure shared mechanisms.

## Limitations

Most importantly, the analytical framework adopted here was primarily exploratory. The aim was to systematically evaluate patterns across multiple data modalities rather than to optimize a single predictive model or to establish mechanistic links between neurobiological alterations and behavioral symptom expression. While the present findings identify which modalities carry predictive information, they do not speak to the directionality or causal structure of brain-behavior relationships in binge-type EDs. This approach provides a comprehensive overview of modality-specific contributions but prohibits causal or mechanistic inference. The observed associations should therefore be viewed as hypothesis-generating and require replication in independent samples. Second, although the present sample is comparatively large for a multimodal neuroimaging study (Szucs and Ioannidis, 2020), it remains moderate for ML analyses and may have lacked power to detect small but meaningful effects (Poldrack et al., 2020; Marek et al., 2022). Third, as the severity indices used here capture real-world symptom expression which varies considerably over time (Peterson et al., 2012; Schaefer et al., 2020), they may also capture related behavioral dynamics we have not directly assessed (e.g., treatment seeking or dieting). Repeated or longitudinal designs would be better equipped to model such fluctuations. Fourth, while integrating multiple data modalities increases ecological validity, it also introduces methodological complexity and a higher risk of overfitting (Varoquaux et al., 2017). Despite rigorous cross-validation and the use of baseline models to mitigate bias, residual confounding, particularly from metabolic factors linked to body weight, cannot be fully excluded (Dinga et al., 2020). Fifth, we did not perform extensive hyperparameter tuning of the machine learning models. While this choice was made to reduce the risk of overfitting in a moderate-sized sample and to maintain comparability across the large number of models tested, future studies with larger datasets may benefit from nested cross-validation and systematic hyperparameter optimization. Finally, cross-diagnostic comparisons in binge-type EDs are complicated by differences in diagnostic criteria and severity definitions for BN and BED (American Psychiatric, 2013). To enable dimensional analyses, we derived composite severity scores aligning key symptom domains. Nevertheless, frequent diagnostic crossovers and symptom fluidity (Tozzi et al., 2005; Eddy et al., 2008) introduce uncertainty into diagnostic labels and thereby constrain the attainable accuracy of classification models. Obesity introduces additional neurobiological variance overlapping with binge-eating-related alterations (Boswell et al., 2021; Saruco and Pleger, 2021; Sutton et al., 2022) and across data modalities in general (Nimptsch et al., 2019; Ju et al., 2025).

## Conclusion

Taken together, our findings suggest that the reduced benefit of multimodal integration reflects the heterogeneous and multi-layered nature of binge-type EDs rather than a limitation of the modeling approach. The modality-specific patterns identified here reflect associations derived from a predictive modeling framework and do not speak to the directionality or causal structure of brain-behavior relationships in binge-type EDs. Findings should therefore be interpreted as hypothesis-generating rather than mechanistic. We found that distinct modalities captured complementary aspects of binge-type EDs. Brain functional connectivity provided the most consistent and generalizable information, reflecting trait-related aspects of brain organization associated with symptom burden, whereas task-based and peripheral biomarkers contributed variance specific to diagnostic distinctions, particularly between BN and BED. This pattern indicates that predictive performance depends on aligning the temporal and biological characteristics of each modality with the clinical feature of interest. For example, measures indexing enduring neural organization may be most relevant for chronic symptom load, whereas peripheral or task-based indices may better capture diagnosis-specific processes such as altered reward sensitivity or metabolic strain. Moving forward, combining multimodal and longitudinal approaches will be key to disentangling state from trait variability, improving individual-level prediction, and advancing precision models of EDs that can ultimately inform diagnosis, prognosis, and personalized intervention.

## Data Availability

The datasets presented in this study can be found invonline repositories. The names of the repository/repositoriesvand accession number(s) can be found at: https://github.com/LenaRo09/NeuroBED_ML.
