# Supplementary material for: Multimodal machine learning reveals neurobiological signatures of binge-type eating disorders
Source: Front Neurosci. 2026 Apr 13;20:1803154. doi: 10.3389/fnins.2026.1803154 (PMC13111282; doi:10.3389/fnins.2026.1803154)
Supplement: Supplementary file 1 [file Data_Sheet_1.pdf]

Distinct and Transdiagnostic Neurobiological Signatures of Binge-Type Eating Disorders

Lena Rommerskirchen, MSc[1]; Mandy Skunde, PhD[12]; Martin Bendszus, MD, PhD[3]; Wolfgang Herzog, MD[1], Hans-Christoph Friederich, MD[14]; Joe J. Simon, PhD[14]

Affiliations:

[1]Department of General Internal Medicine and Psychosomatics, Centre for Psychosocial Medicine, University Hospital Heidelberg, Heidelberg, Germany

[2]Institute of Pathology, University Hospital Heidelberg, Heidelberg, Germany.

[3]Department of Neuroradiology, University Hospital Heidelberg, Heidelberg, Germany

[4]DZPG (German Centre for Mental Health – Partner Site Heidelberg/ Mannheim/ Ulm)

Author for correspondence:

Joe J. Simon, Ph.D.

Centre for Psychosocial Medicine, General Internal Medicine and Psychosomatics, University Hospital Heidelberg, Im Neuenheimer Feld 410, 69120 Heidelberg

Tel.: +49 (0)6221 56-38667

Fax: +49 (0)6221 56-5988

Joe.simon@med.uni-heidelberg.de

A. Procedure

All participants were tested individually in a single session between 9:00 a.m. and 2:00 p.m. They were instructed to come to the clinic without having breakfast and to refrain from consuming alcoholic drinks for 24 hours prior to the experiment. Upon arrival, participants received a light standardized breakfast containing approximately 550 kcal at 9:00 a.m. After breakfast, all participants completed the Structured Clinical Interview for DSM-V (SCID[1]), followed by a series of self-report questionnaires and neuropsychological tasks. Eating behavior was assessed using the Dutch Eating Behavior Questionnaire (DEBQ[2]), which consists of subscales for restrained eating, emotional eating, and external eating. Eating disorder psychopathology was assessed using the Eating Disorder Examination Questionnaire (EDEQ[3]), which includes subscales for restraint eating, eating concern, weight concern and shape concern. Furthermore, depressive symptoms were measured with the Beck Depression Inventory-II (BDI-II[4]), and impulsivity was assessed using the Barratt Impulsiveness Scale (BIS-11[5]), which includes attentional, motor, and non-planning subscales. Participants also completed the State and Trait versions of the General Food Craving Questionnaire (G-FCQ[6]). Following this, participants performed neuropsychological tests assessing inhibitory control (SST, Stop Signal Task, designed to assess motor inhibition[7]), cognitive control related to response inhibition (RSS, Response Set Shifting task[8]), cognitive flexibility related to the updating, representation, and maintenance of frequently changing task rules (CSS, Cued Set Switching[9]), short-term auditory memory and working memory (WMS-DS, Wechsler Memory Scale Digit Span subtest[10], and premorbid verbal intelligence (MWT-B, Multiple-Choice Vocabulary Intelligence Test[11]). At 12:00 p.m., MRI scanning was performed, corresponding to lunchtime for most participants. Prior to the scan, the experimental procedure was

explained, and participants were trained in the experimental paradigms (Supplement B). Hunger and mood ratings were also obtained before the fMRI measurement.

B. Paradigmns and Univariate Analyses

fMRI tasks

Two experimental tasks were employed in this study. First, we used an incentive delay task to assess neuronal processing during both the anticipation and receipt of monetary and food-related rewards. Specifically, during the monetary incentive delay task (MID), participants could win money throughout the experiment, which they were able to exchange for real money immediately after the MRI session. During the food incentive delay task (FID), participants could win "snack points" during the experiment, which they could exchange for sweet and salty snacks, as well as beverages and fruit, immediately after the MRI measurement. Using abstract representations rather than actual foods allowed us to directly compare food-related with monetary rewards, and also helped to avoid variance caused by individual differences in food preferences. The task was divided into four runs with 55 trials each, featuring either monetary or food-specific rewards, and was presented in a counterbalanced fashion across participants. Further details regarding the task can be found in our previous publications utilizing this paradigm[12-16]. Second, we employed a Go-Nogo task to assess neuronal processing during response inhibition to abstract and food-related stimuli. Specifically, participants were instructed to respond as quickly and accurately as possible to frequent target stimuli (“go” trials; squares in the abstract version or household items in the food-related version) and to inhibit any response to rare nontarget stimuli (“no-go” trials; circles in the abstract version or individually selected food pictures in the food-related version). Prior to scanning, participants selected eight

of their favorite foods from a set of 85 custom-made pictures depicting high-calorie sweet and savory foods. The eight pictures of household items were identical for all participants. After the scan, participants rated the individually selected food pictures and the household items on arousal, valence, incentive salience ("wanting"), hedonic value ("liking"), and urge to binge eat (foods only). The task was divided into two runs, each with eight blocks (40 trials each, either abstract or food-related stimuli), presented in a counterbalanced sequence across participants. Participants viewed visual stimuli on a projection screen via a mirror fixed to the head coil and responded with the right hand using a button box (see also[17]).

fMRI acquisition

Images were collected using a 3-T Siemens Trio MRI scanner (Siemens Medical Solutions, Erlangen, Germany) equipped with a standard 32-channel head coil. For the incentive delay tasks, participants performed four functional runs lasting 9.3min each, with 280 volumes per run. The Go/No-Go task consisted of two runs, each lasting 10.1min with 303 volumes per run. Before the experimental tasks began, a resting-state fMRI measurement was performed, lasting 5.4min with 162 volumes. For both tasks and the resting-state measurement, T2*-weighted, single-shot EPI images were collected, with 30 interleaved axial slices covering the whole brain at a thickness of 4mm and a 1-mm interslice gap. For the incentive delay task, slices were acquired at a 10° oblique angle relative to the AC-PC axis to minimize susceptibility artifacts in the orbitofrontal cortex; for the Go/No-Go task and resting-state measurement, slices were acquired parallel to the anterior and posterior commissure. Imaging parameters were as follows: repetition time (TR)=2000ms, echo time (TE)=30ms, flip angle=80°, field of view=192×192mm², resulting in an in-plane resolution of 3×3×4mm³. Finally,

high-resolution T1-weighted MPRAGE anatomical images were acquired (192 slices; voxel size=1×1×1mm³; TR=1570ms; TE=2.63ms; flip angle=9°).

fMRI-preprocessing

Pre-processing of fMRI data for the functional tasks are detailed in the respective publications[14,17]. In brief, for both tasks, functional MRI data were preprocessed and analyzed using SPM8. The first four scans of each run were discarded to allow for magnetic field equilibration. Images were manually reoriented to the AC-PC line, checked for artifacts, slice time corrected, realigned (motion limited to ±3–4mm translation and ±3° rotation), and coregistered (T1 to mean T2* image). Functional and anatomical images were normalized to MNI space (ICBM152; functional voxel size: 3mm³ or 3×3×4mm³; anatomical voxel size: 1mm³), and functional images were smoothed with an 8-mm FWHM Gaussian kernel. For the incentive delay task, images were also unwarped and a 128-s high-pass filter was applied. For the Go/No-Go task, a 256-s high-pass filter was used.

fMRI first level analysis and univariate analysis of fMRI tasks

Incentive Delay Task

At the first-level analysis, a general linear model (GLM) was constructed including separate regressors for the anticipation of high reward (EUR 1 or 10 snack points), anticipation of low reward EUR 0.20 or 2 snack points), and no reward (EUR 0 or 0 snack points) and outcome phases (receipt or omission of high, low, and no rewards), convolved with a gamma-variate function. Targets and error trials were modeled as regressors of no interest. Contrasts of interest specifically compared the anticipation of high reward versus no reward, and the receipt of high reward versus no reward, to maximize activation in reward-sensitive neural circuits. At the second-level analysis, individual contrasts were subjected to random-effects analyses to assess within-group

activations via one-sample t-tests, and between-group differences using two-sample t-tests. Analyses included small-volume correction in predefined reward-related regions of interest (ROIs) and whole-brain analyses, both applying cluster-level family-wise error (FWE) correction. Mean percent signal change was extracted from these regions and correlated with eating-related psychometric assessments. For further details please refer to the previous publications[14,15].

Go/No-Go Task

For the first-level analysis, four regressors modeled correct responses to go and no-go trials separately for abstract stimuli (go: squares; no-go: circles) and food-related stimuli (go: household items; no-go: individualized food pictures). Incorrect trials, instructions, rest periods, and motion parameters were modelled as regressors of no interest. Contrasts of interest were defined as no-go versus go trials separately for abstract stimuli (circles vs. squares) and food-related stimuli (food vs. household items) to isolate neural correlates of response inhibition. At the second-level analysis, within-group activations for each contrast (abstract and food-related) were evaluated via one-sample t-tests, and between-group differences were assessed using two-sample t-tests. Corrections for multiple comparisons were applied using a cluster-level FWE correction with predefined significance thresholds. Additionally, small-volume correction was applied to specific anatomical regions of interest (e.g., right dorsal striatum). Signal changes from significant clusters were extracted for further correlation analyses with clinical or behavioral measures. For further details please refer to the previous publication[17].

For both tasks, the MarsBaR[18] toolbox was used to extract signal change for all 136 ROIs of the AAL3 atlas[19] (excluding the thalamic nuclei which were replaced by the AAL2 partition), corresponding to the respective regressors of interest. Specifically, for

the incentive delay task, signal change corresponding to the 3 different expectation phases (high, low and zero), and 4 different outcome phases (high win and high no win, low win and low no win, zero) for both monetary and food-related rewards were extracted, and for the Go/No-Go task, signal change corresponding to go-trials (food and abstract) and no-go trials (food and abstract) were extracted.

Resting state analysis

Analyses of rsfMRI data were performed using the CONN-toolbox[20]. Functional and anatomical data were preprocessed using a modular preprocessing pipeline[21] including band-pass filtering, outlier detection, and ROI extraction. BOLD signal timeseries were bandpass filtered between 0.01 Hz and 0.1 Hz. Potential outlier scans were identified using ART[22] as acquisitions with framewise displacement above 0.9 mm or global BOLD signal changes above 5 standard deviations[23], and a reference BOLD image was computed for each subject by averaging all scans excluding outliers. The average BOLD signal as well as the largest principal components within regions of the AAL3 atlas[19] were extracted in percent signal change (PSC) units. Last, anatomical data were segmented into grey matter, white matter, and CSF tissue classes using SPM unified segmentation and normalization algorithm with the default IXI-549 tissue probability map template. In addition, functional data were denoised including the regression of potential confounding effects characterized by white matter timeseries (5 CompCor noise components), CSF timeseries (5 CompCor noise components), motion parameters and their first order derivatives (12 factors), outlier scans (below 67 factors), session effects and their first order derivatives (2 factors), and linear trends (2 factors) within each functional run, followed by bandpass frequency filtering of the BOLD timeseries between 0.008 Hz and 0.09 Hz[21]. CompCor (Behzadi et al., 2007) noise components within white matter and CSF were estimated

by computing the average BOLD signal as well as the largest principal components orthogonal to the BOLD average, motion parameters, and outlier scans within each subject's eroded segmentation masks. Following this, ROI-to-ROI connectivity (RRC) matrices were estimated characterizing the functional connectivity between each pair of regions among 166 ROIs from the AAL3 atlas[19]. Functional connectivity strength was represented by Fisher-transformed bivariate correlation coefficients from a general linear model, estimated separately for each pair of ROIs. In order to compensate for possible transient magnetization effects at the beginning of each run, individual scans were weighted by a step function convolved with an SPM canonical hemodynamic response function and rectified. For each participant, the upper triangle of the ROI-to-ROI connectivity matrix was extracted.

Parcel-wise resting-state pre-analysis

To reduce the dimensionality of the resting-state feature space, we adopted a parcel-wise functional connectivity (FC) approach following Weis et al.[24] rsfMRI data were parcellated with the AAL3 atlas (166 regions). For each subject and parcel, we calculated the mean BOLD signal as the parcels time series computed Pearson correlations with every other parcel, excluding self-connections (diagonal).

For the parcel-wise screen, we re-ran the same classification and regression pipelines used in the main analysis, but trained them separately for each parcel using that parcel's row-wise connectivity vector (165 features). Across all targets and parcels this yielded 4,482 models. Parcels were ranked by cross-validated performance, and the top 10 per target were retained for downstream analyses. This reduced the candidate feature space from the full connectome (166 × 166; 13,695 unique undirected edges) to a focused set of parcel-centric profiles (10 parcels per target, each with 165 connections).

Finally, we conducted an exploratory enrichment analysis to test whether top parcels clustered by functional systems and whether these distributions differed across targets.

VBM analysis

Structural T1-weighted MRI images were preprocessed using SPM12 following the standard voxel-based morphometry (VBM) protocol. First, using the unified segmentation approach, all anatomical images were bias corrected to compensate for intensity inhomogeneities and improve tissue classification. Subsequently, the bias-corrected images were segmented into gray matter tissue images and spatially normalized to the Montreal Neurological Institute (MNI) standard space using the nonlinear deformation fields derived during segmentation. The normalized gray matter maps were resliced and smoothed using an 8mm isotropic Gaussian kernel. AAL3 ROIs were resliced to match the native T1-weighted anatomical image resolution and binarized. MarsBaR[18] was then used to extract the mean raw gray matter volume from anatomical T1-weighted AAL3 ROIs.

C. Machine Learning Framework

MRI feature extraction

Across all MRI modalities, the AAL3 atlas (166 cortical/subcortical ROIs[19]) was used for brain parcellation. To avoid very small task fMRI parcels of the thalamic nuclei, AAL3 thalamic nuclei were replaced by the coarser bilateral thalamus ROIs from AAL2 for the task-based analyses. Signal change for all specified ROIs was then extracted using the the MarsBaR toolbox[18]. For structural MRI, AAL3 ROIs were resliced to each participant’s native T1-weighted space and binarized prior to extracting the mean raw gray matter volume (GMV) from anatomical T1-weighted AAL3 ROIs.

Functional connectivity of rsfMRI was computed as the Pearson correlation between the mean time series of every pair of AAL3 ROIs (166 × 166). Correlations were Fisher-z transformed. To compensate for potential transient magnetization effects at run onset, individual scans were weighted by a step function convolved with the SPM canonical hemodynamic response function and rectified during model estimation. For each participant, the upper triangle of the ROI-to-ROI matrix (excluding the diagonal; 13.695 unique connections) was retained as the rsfMRI feature vector. To reduce the dimensionality of the resting-state feature space, we adopted a parcel-wise functional connectivity (RSFC) approach following Weis et al. ([24], see Supplementary Material B). For each target-sample combination, we first trained a separate set of models using each parcel's row-wise connectivity vector (165 features). Across all targets and parcels this yielded 4,482 additional models. Parcels were then ranked by cross-validated performance, and the top 10 parcels for each target-sample combination were retained for downstream analyses. This reduced the candidate feature space from the full connectome to a focused set of parcel-centric profiles (10 parcels per target, each with 165 connections). A detailed description of the fMRI employed tasks, fMRI acquistion procedures, preprocessing of fMRI-data and first level analysis and univariate analysis of fMRI tasks as well as resting-state pre-analyses are provided in the Supplement. We also provide a data exploration notebook with descriptive feature analyses.

Peripheral Blood Biomarkers

Peripheral blood parameters included metabolic and endocrine markers implicated in appetite and weight regulation. Specifically, glucose, γ-glutamyltransferase (GGT), triglycerides, total cholesterol, progesterone, and estradiol (E2) were extracted for each participant. These markers have been consistently linked to the regulation of

energy intake and body weight, with glucose, lipid, and liver enzyme metabolism reflecting short- and long-term metabolic adaptations, and sex hormones modulating these processes via their influence on energy homeostasis[25-30]. Assessment of peripheral blood parameters was performed at the central laboratory of the University Clinic Heidelberg. Plasma glucose concentrations were assessed on a Siemens Advia 2400 device using the hexokinase method. Serum based GGT values were determined using a kinetic photometric assay (Szasz/Persijn method, IFCC-standard). Triglyceride and total cholesterol concentrations were quantified using an enzymatic colorimetric GPO-PAP method. Progesterone concentrations were determined using a quantitative immunoassay based on chemiluminescent detection and estradiol concentrations were measured using a quantitative immunoassay with chemiluminescent detection on a ZMOM analyzer.

Preprocessing and confound handling

Prior to modeling, all features were screened for implausible values using feature-wise z-scores, with a conservative threshold of $|z|>10$. Below that threshold, values were retained if they were physiologically plausible (e.g., markedly elevated glucose in individuals with obesity). Obvious measurement errors, particularly in blood markers, were removed. For each participant, features with >50% missing features were excluded from further analyses. Two subjects were removed beforehand because most data modalities were missing. Since BMI is closely related to many imaging, behavioral, and blood measures, we removed BMI-related variance when weight was expected to influence the target or when the contrast was not designed to control BMI (i.e. BN vs BED). To focus on BMI-independent signal for regression targets, BMI was not included as a target for eating- and weight-related outcomes (Eating-specific, Eating-unspecific, Binge-specific, Weight-fluctuation and monitoring). BMI was not

removed for Disease-unspecific (e.g., depressive symptoms) to avoid over-adjustment.

Dimensionality Reduction

Dimensionality reduction was performed using principal component analysis (PCA) as implemented in sklearn.decomposition.PCA[31]. PCA was fitted within each training fold to avoid data leakage and subsequently applied to the held-out test set. Before PCA, features were median-imputed (sklearn.impute.SimpleImputer(strategy='median')) and scaled using a robust scaler (sklearn.preprocessing.RobustScaler()).

The number of retained components $\boldsymbol{n}_{\text{PCA}}$ was determined dynamically for each training set as:

$$\boldsymbol{n}_{\text{PCA}} = \boldsymbol{min}\,(\boldsymbol{n}_{\text{max}}, \boldsymbol{p}, \lfloor \boldsymbol{\alpha} \cdot \boldsymbol{p} \rfloor, \boldsymbol{n}_{\text{train}} - \mathbf{1})$$

where:

$\boldsymbol{n}_{\text{max}} = \mathbf{100}$ is the global upper bound,

$\boldsymbol{p}$ is the number of available features,

$\boldsymbol{\alpha} = \mathbf{0.8}$ is the target proportion of retained variance, and

$\boldsymbol{n}_{\text{train}}$ is the number of training samples in the current outer cross-validation fold.

Thus, PCA retained approximately 80% of the original feature variance, constrained by feature dimensionality and sample size. For single-modality models, PCA was applied once across all features of that modality.

For multimodal models, PCA was performed separately per modality subset (e.g., behavioral, structural MRI, task-fMRI, resting-state fMRI, blood) with an identical $\boldsymbol{n}_{\text{PCA}}$ shared across modalities, ensuring comparable feature compression before concatenation (“late fusion”). No whitening was applied (whiten=False). All PCA steps

were implemented using julearn's PipelineCreator, ensuring consistent preprocessing and fold-wise isolation within the cross-validation framework.

Functional-group enrichment of top RSFC features

To assess whether the 10 most predictive parcels tended to cluster within specific functional systems-and whether these distributions differed by prediction target, we performed an exploratory hypergeometric test with BH FDR correction[32] on the top 10 parcels selected per target from the parcel-wise resting-state pipeline. Parcels were assigned to functional systems to ease interpretability (e.g., DMN, Frontal, Thalamus etc., see Supplementary Excel file). The number of parcels for each grouping was calculated separately for each target. Expected counts under the null were computed proportional to system size within the candidate set:

$$expected\_g = 10 * (n\_g \ / \ N)$$

As effect size, we report the enrichment ratio (Observed/Expected) and, in sensitivity analyses, a rank-weighted variant where parcels contributed inverse-rank weights (10 for rank-1 … 1 for rank-10) instead of unit counts. Classification and regression targets were corrected independently.

D. Results

Functional-group enrichment of top RSFC features

Across the **classification targets**, two contrasts showed significant functional-system enrichment after FDR correction. For BED vs BN, Salience-network parcels were strongly overrepresented among the top predictive parcels (5/10; 7.1× expected; $q=7.5\times10^{-4}$). For patients vs controls, parcels assigned to the Default Mode Network (DMN) were enriched (3/10; 6.4× expected; q=0.050). No other diagnostic contrasts exhibited significant system-level enrichment after correction. For the **regression targets**, disease-unspecific severity (BDI) showed significant enrichment for Visual (6/10; 7.3× expected; $q=8.7\times10^{-5}$), Frontal (5/10; 3.9×; q=0.023), and Cerebellar (5/10; 3.3×; q=0.042) systems. BN-specific severity showed significant enrichment for Temporal parcels (3/10; 6.4×; q=0.028) as well as Limbic and Striatal parcels (3/10 each; 4.6–5.1× expected; q≈0.046–0.049). No additional severity targets demonstrated significant enrichment after FDR correction. Results of the parcel-wise RSFC pre-analysis are provided in Supplementary Tables 2-7 and Supplementary Figure 1.

E. Supplementary Figures

Supplementary Figure 1. Top predictive resting-state parcels by group and contrast.

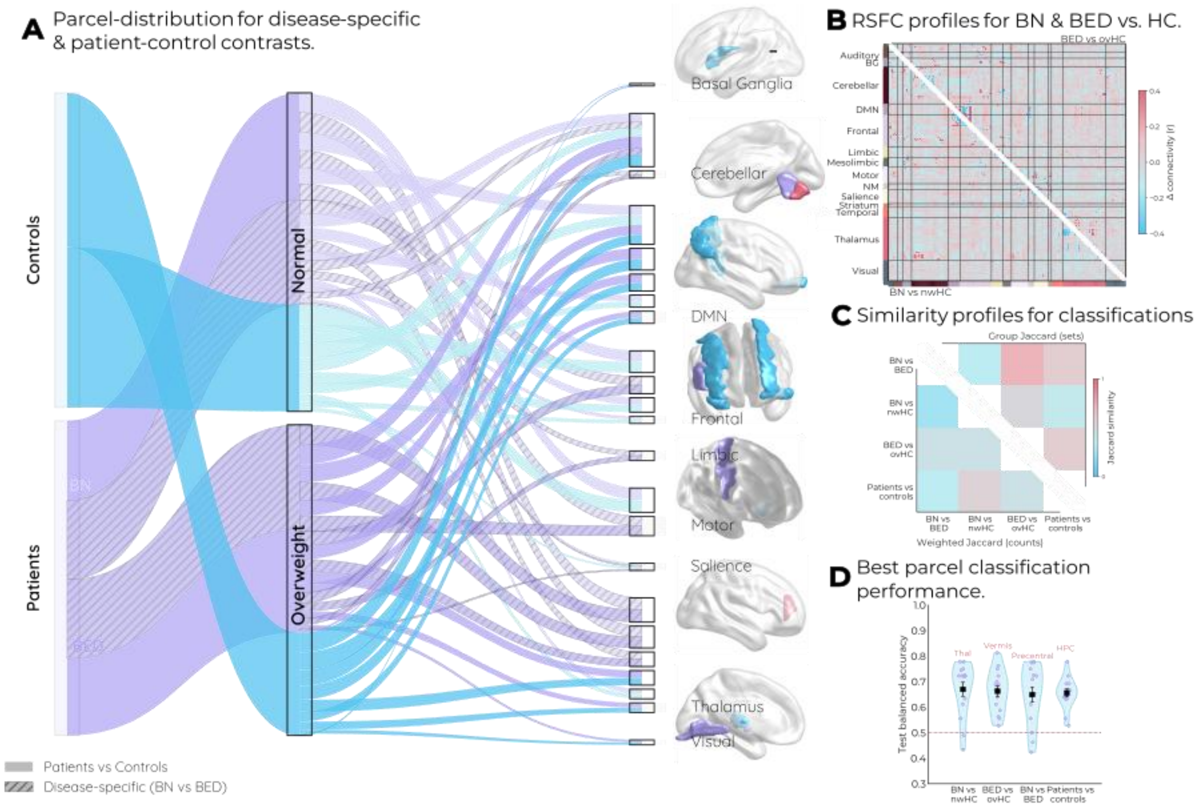

(A). Nested Sankey showing how the top 10 predictive parcels (AAL3) are distributed across Disease-status (patient vs. controls; left) and weight status (normal vs. overweight; right). Right: individual parcels ordered and grouped by functional systems (patients: purple; controls: blue; both: red). Hatched bands mark disease-specific contrasts (BN vs BED); solid band mark patient-vs-control contrasts. Band labels on the right indicate the functional systems in which the most predictive parcels cluster. (**B).** RSFC map for BN vs nwHC (lower triangle) and BED vs owHC (upper). Colors indicate direction and magnitude of Δ connectivity (blue = higher in BN/BED, red = higher in controls). (**C).** Similarity of predictive parcels across targets. Pairwise overlap of top rsfMRI parcels between classification targets. The upper triangle shows the weighted Jaccard (overlap of normalized parcel importance weights), and the lower triangle shows the group-threshold Jaccard (overlap after binarizing parcels that met the group threshold across folds). Warmer colors indicate greater similarity; the diagonal is masked. Overall, overlap is modest and target-specific rather than global (**D).** Test balanced accuracy for the best single parcel per classification target (cross-validation folds shown as points; black squares = mean ± SEM). Dashed line at 0.5 denotes chance.

Supplementary Figure 2. Mean test R² for all group × outcome × single-modality combinations.

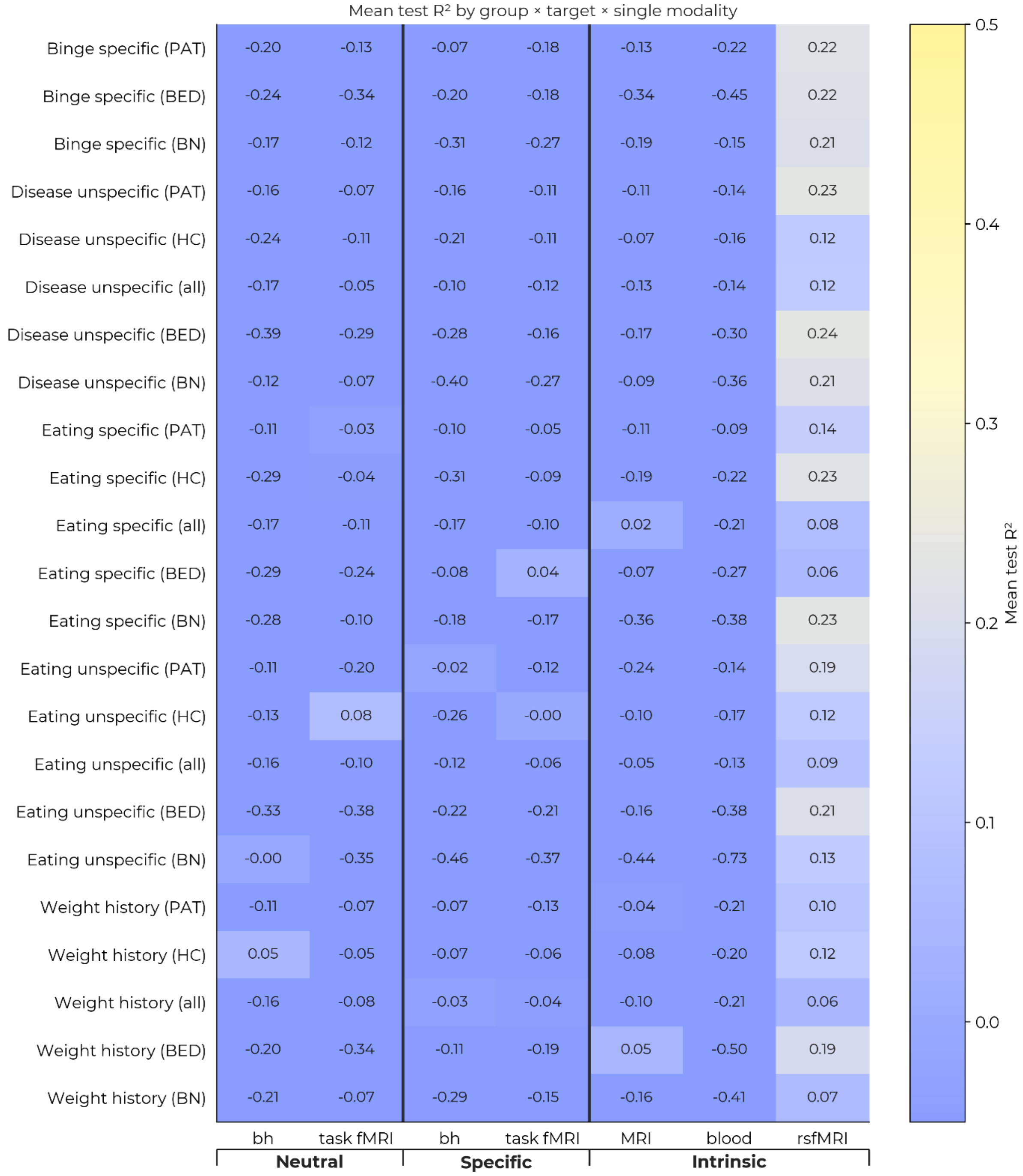

Each cell shows the mean cross-validated R² on held-out folds for a single-modality predictor set (columns) and a specific outcome target within a group (rows); color encodes R². Vertical lines separate modality families (Neutral, Specific, Both) as defined in Methods; within families, columns denote the individual modalities. Values near 0 indicate performance comparable to predicting the test-fold mean, and negative values indicate worse-than-mean performance. Abbreviations: BN = bulimia nervosa; BED = binge-eating disorder; PAT = patients (all clinical groups combined); HC = healthy controls; bh = behavioral measures; task fMRI = task-based functional MRI; rsfMRI = resting-state fMRI; MRI = structural MRI (gray-matter morphometry); blood = peripheral blood biomarkers.

F. Supplementary Tables

Supplementary Table 1. Demographic and Clinical Characteristics of Participants

Variable	BED (N=26) M (SD)	BN (N=29) M (SD)	owHC (N=28) M (SD)	nwHc (N=27) M (SD)	*BED vs. BN* *P*-Value[b]
Age, mean (SD), y	38.54 (13.95)	27.45 (10.55)	38.00 (10.85)	25.74 (5.25)	*0.002*
BMI, mean (SD), kg/m^2	32.71 (4.61)	21.33 (2.99)	34.02 (4.50)	21.85 (1.85)	*<0.001*
nr binges, mean (SD)	2.63 (1.79)	3.83 (2.58)	na	na	0.053
Frequency of self-weighing, mean (SD), per week	3.20 (1.35)	4.10 (2.18)	4.61 (1.57)**	5.48 (1.48)*	0.078
nr gains plus 5 kg, mean (SD)	6.42 (4.06)	4.10 (3.92)	2.75 (3.33)***	0.93 (1.57)***	*0.036*
nr gains plus 6 to 10 kg, mean (SD)	2.38 (2.10)	1.45 (2.28)	0.93 (0.86)**	0.56 (1.31)	0.12
nr gains plus11 to 20 kg, mean (SD)	0.69 (0.84)	0.62 (0.82)	0.32 (0.67)	0.04 (0.19)***	0.75
nr gains plus 21 to 30 kg, mean (SD)	0.23 (0.59)	0.17 (0.47)	0.07 (0.26)	0.00 (0.00)	0.684
nr gains plus 31 to 40 kg, mean (SD)	0.08 (0.27)	0.00 (0.00)	0.00 (0.00)	0.00 (0.00)	0.133
nr gains plus 40 kg, mean (SD)	0.04 (0.20)	0.00 (0.00)	0.11 (0.42)	0.00 (0.00)	0.295
DEBQ: restrained eating, mean (SD)	19.27 (7.05)	28.83 (7.63)	15.39 (6.97)*	10.19 (7.19)***	*<0.001*
DEBQ: emotional eating, mean (SD)	28.00 (9.10)	28.53 (7.92)	13.61 (9.24)***	6.26 (4.05)***	0.817
DEBQ: external eating, mean (SD)	26.12 (7.12)	25.17 (7.18)	20.54 (6.37)**	18.33 (6.92)***	0.627
G-FCQ: state, mean (SD)	40.40 (14.84)	39.55 (13.49)	33.21 (13.49)	33.00 (10.06)*	0.827
G-FCQ: trait, mean (SD)	83.04 (19.37)	83.59 (15.71)	57.54 (17.60)***	43.59 (11.78)***	0.909
EDEQ: restraint, mean (SD)	12.88 (7.39)	17.07 (7.95)	7.18 (5.53)**	1.81 (2.25)***	0.051
EDEQ: eating_concern, mean (SD)	15.28 (6.79)	17.69 (6.14)	3.64 (4.21)***	0.41 (0.64)***	0.177
EDEQ: weight_concern, mean (SD)	20.32 (4.69)	19.62 (8.46)	10.71 (5.75)***	2.26 (2.80)***	0.715
EDEQ: shape_concern, mean (SD)	36.64 (7.36)	34.38 (12.23)	21.14 (10.62)***	5.93 (5.42)***	0.424
EDEQ: total_score, mean (SD)	100.04 (23.13)	112.14 (24.66)	76.54 (19.67)***	64.93 (13.58)***	0.07
BDI, mean (SD)	23.65 (12.41)	24.79 (12.49)	8.61 (7.48)***	3.11 (2.62)***	0.736

Mean values and standard deviations (SD) were rounded to two decimal points; p-values were rounded to three decimal points. P-values result from two-sample, two-tailed t-tests comparing BED and BN groups. Asterisks in the columns owHC and nwHC indicate significant differences based on separate two-sample t-tests comparing owHC with BED and nwHC with BN, respectively. Significance levels are marked as: *$p < 0.05$, **$p < 0.005$, ***$p < 0.001$. BED = Binge Eating Disorder; BN = Bulimia Nervosa; owHC and nwHC = Healthy control groups matched to BED and BN, respectively; M = Mean value; SD = Standard deviation; N = Number of participants; BMI = Body Mass Index; VAS = Visual Analogue Scale; DEBQ = Dutch Eating Behavior Questionnaire; G_FCQ = General Food Cravings Questionnaire; EDEQ = Eating Disorder Examination Questionnaire; BDI = Beck Depression Inventory.

Supplementary Table 2. Parcel-wise rsfMRI pre-analysis for classification targets.

Target	Confounder	ROIs	Range	Metric
BED vs BN	BMI	Cingulate, Frontal, Olfactory, Red Nucleus, Thalamus, Vermis	0.568–0.649	bACC
BED vs owHC	None	Caudate, Cingulate, Frontal, Occipital, Other, Parietal, Thalamus, Vermis	0.578–0.663	bACC
BN vs nwHC	None	Cingulate, Frontal, Parietal, Thalamus, Vermis	0.624–0.670	bACC
Patients vs Controls	None	Cingulate, Frontal, Hippocampus, Parietal, Thalamus, Vermis	0.584–0.655	bACC

For each diagnostic contrast, we list the confound included in the model (if any), the dominant region families among the top-performing parcels (ROI-to-functional grouping can be found in the Supplementary Excel File), and the Range of parcel-level balanced accuracy (bACC) across those parcels (min–max). These results highlight recurring cingulo-frontal–thalamic and cerebellar (vermis) contributions across contrasts. Abbreviations: BED=binge eating disorder; BN=bulimia nervosa; owHC=overweight healthy controls matched to BED; nwHC=normal=weight healthy controls matched to BN; BMI=body mass index; ROI(s)=region(s) of interest; rsfMRI=resting=state functional MRI; bACC=balanced accuracy.

Supplementary Table 3. Parcel-wise rsfMRI pre-analysis for eating unspecific severity prediction

Target	Group	Conf.	ROIs	Range	Metric
Eating unspecific	All	BMI	Cerebellum, Hippocampus, Insula, Olfactory, Other, Parietal, Thalamus	0.016–0.048	R^2
	BED	BMI	Cerebellum, Cingulate, Frontal, Locus Coeruleus, Occipital, Temporal, Thalamus	0.036–0.198	R^2
	BN	BMI	Amygdala, Frontal, Locus Coeruleus, Occipital, Other, Rectus, Temporal, Thalamus	-0.035–0.109	R^2
	PAT	BMI	Amygdala, Frontal, Locus Coeruleus, Occipital, Other, Rectus, Temporal	0.051–0.137	R^2
	HC	BMI	Cerebellum, Frontal, Insula, Olfactory, Other, Red Nucleus, Thalamus, Vermis	0.026–0.067	R^2

The score combines the DEBQ and FCQ subscales. R^2 values may be negative when predictions underperform a simple mean-prediction baseline on the validation fold. Abbreviations: BED=binge eating disorder; BN=bulimia nervosa; DEBQ = Dutch Eating Behavior Questionnaire (Grunert, 1989); FCQ = Food Choice Questionnaire (Nijs et al., 2007); PAT=patients (BED + BN); HC=healthy controls; All=all participants combined; BMI=body mass index; ROI(s)=region(s) of interest; rsfMRI=resting-state functional MRI; LC=locus coeruleus; Rectus=gyrus rectus; Vermis=cerebellar vermis; Other=parcels not covered by the listed families.

Supplementary Table 4. Parcel-wise rsfMRI pre-analysis for disease unspecific severity prediction.

Target	Group	Conf.	ROIs	Range	Metric
Disease unspecific	All	None	Cerebellum, Cingulate, Frontal, Parietal, Temporal, Vermis	0.009–0.111	R^2
	BED	None	Cingulate, Occipital, Temporal, Thalamus	0.105–0.214	R^2
	BN	None	Occipital, Other, Parahippocampal, Parietal	0.083–0.167	R^2
	PAT	None	Frontal, Occipital, Other, Putamen	0.080–0.194	R^2
	HC	None	Cerebellum, Cingulate, Other, Parietal, Temporal, Thalamus, Vermis	-0.063–0.039	R^2

Disease-unspecific severity score reflects general depressive symptoms as measured by the BDI questionnaire. R^2 values may be negative when predictions underperform a simple mean-prediction baseline on the validation fold. Abbreviations: BED = binge-eating disorder; BN = bulimia nervosa; PAT = patients (all clinical groups combined); HC = healthy controls; ROIs = regions of interest; R^2 = coefficient of determination.

Supplementary Table 5. Parcel-wise rsfMRI pre-analysis for disease weight fluctuations and monitoring prediction.

Target	Group	Conf.	ROIs	Range	Metric
Weight Fluctuations and Monitoring	All	BMI	Cerebellum, Olfactory, Other, Temporal, Thalamus, Vermis	-0.007–0.037	R^2
	BED	BMI	Cingulate, Frontal, Pallidum, Parietal, Red Nucleus, Temporal, Thalamus, Vermis	0.058–0.110	R^2
	BN	BMI	Cerebellum, Cingulate, Hippocampus, Occipital, Other, Parietal, Raphe	0.002–0.085	R^2
	PAT	BMI	Cerebellum, Cingulate, Frontal, Other, Parietal, Temporal, Thalamus	0.004–0.065	R^2
	HC	BMI	Caudate, Cingulate, Frontal, Other, Pallidum, Temporal	0.025–0.057	R^2

The score reflects lifetime weight fluctuations and monitoring behavior. R^2 values may be negative when predictions underperform a simple mean-prediction baseline on the validation fold. Abbreviations: BED = binge-eating disorder; BN = bulimia nervosa; PAT = patients (all clinical groups combined); HC = healthy controls; ROIs = regions of interest; R^2 = coefficient of determination.

Supplementary Table 6. Parcel-wise rsfMRI pre-analysis for disease eating-specific severity prediction.

Target	Group	Conf.	ROIs	Range	Metric
Eating specific	All	BMI	Cerebellum, Cingulate, Frontal, Locus Coeruleus, Occipital, Other, Temporal, Vermis	-0.001–0.050	R^2
	BED	BMI	Caudate, Cerebellum, Cingulate, Other, Temporal, Thalamus, Vermis	0.028–0.124	R^2
	BN	BMI	Cerebellum, Cingulate, Frontal, Other, Parietal, Putamen, Temporal, Thalamus	0.071–0.150	R^2
	PAT	BMI	Cingulate, Frontal, Hippocampus, Insula, Other, Putamen, Temporal	-0.001–0.153	R^2
	HC	BMI	Cerebellum, Cingulate, Olfactory, Pallidum, Temporal, Thalamus	0.003–0.065	R^2

The score reflects disorder-specific pathology (EDEQ total score). R^2 values may be negative when predictions underperform a simple mean-prediction baseline on the validation fold. Abbreviations: BED = binge-eating disorder; BN = bulimia nervosa; PAT = patients (all clinical groups combined); HC = healthy controls; ROIs = regions of interest; R^2 = coefficient of determination.

Supplementary Table 7. Parcel-wise rsfMRI pre-analysis for binge-specific severity prediction.

Target	Group	Conf.	ROIs	Range	Metric
Binge specific	PAT	BMI	Cerebellum, Cingulate, Frontal, Occipital, Vermis	0.058–0.122	R^2
	BED	BMI	Cingulate, Occipital, Other, Temporal, Thalamus, Vermis	0.035–0.203	R^2
	BN	BMI	Amygdala, Cerebellum, Cingulate, Frontal, Locus Coeruleus, Occipital, Other, Thalamus	0.061–0.143	R^2

The score reflects the number of binge-eating episodes per week. R^2 values may be negative when predictions underperform a simple mean-prediction baseline on the validation fold. Abbreviations: BED = binge-eating disorder; BN = bulimia nervosa; PAT = patients (all clinical groups combined); HC = healthy controls; ROIs = regions of interest; R^2 = coefficient of determination.

Classifications

Supplementary Table 8. Significant above chance performance for single modality classification models

target	counfounds	modality	bACC	*t*	*p*
BN vs BED	BMI	blood	0.88	4.93	<0.001
		Task-fMRI (spec)	0.881	5.608	<0.001
BED vs owHC	None	rsfMRI	0.633	1.946	0.036
BN vs nwHC	None	rsfMRI	0.733	5.713	<0.001
		GMV	0.640	2.308	0.018
Patients vs Controls	None	rsfMRI	0.694	4.308	<0.001

Confound indicates whether BMI–related variance was removed prior to modeling (“BMI”) or not (“None”). All other classification models were not significant. Abbreviations: BN = Bulimia nervosa; BED = Binge-eating disorder; HC = Healthy controls; owHC = Overweight/obese HC; nwHC = Normal-weight HC; rsfMRI = Resting-state functional MRI; Task-fMRI (spec) = Task-based fMRI with disorder-specific (food) stimuli; GMV = Gray-matter volume (structural MRI); BMI = Body mass index.

Supplementary Table 9. Pairwise classification performance across groups and modality combinations.

target	Conf.	modality	stimuli	bACC	*t*	*p*
BN vs BED	BMI	GMV+bh (spec)+blood	specific	0.634	1.931	0.037
		bh (neu)+blood+rsfMRI	neutral	0.663	2.065	0.029
		blood+rsfMRI	intrinsic	0.68	2.646	0.01
		bh (spec)+blood+rsfMRI	specific	0.689	1.848	0.043
		bh (neu)+blood	neutral	0.76	3.221	0.003
		bh (spec)+blood	specific	0.771	3.336	0.002
BED vs owHC	None	GMV+bh (neu)+rsfMRI	neutral	0.612	2.023	0.031
		bh (spec)+blood+rsfMRI	specific	0.615	1.944	0.036
		GMV+bh (spec)+blood+rsfMRI	specific	0.616	1.861	0.042
		bh (spec)+rsfMRI	specific	0.639	2.536	0.012
		GMV+rsfMRI	intrinsic	0.64	3.028	0.005
		GMV+bh (spec)+rsfMRI	specific	0.654	2.124	0.026
		bh (neu)+rsfMRI	neutral	0.655	2.811	0.007
BN vs nwHC	None	GMV+bh (neu)+blood	neutral	0.627	2.243	0.021
		GMV+blood	intrinsic	0.635	2.223	0.022
		bh (spec)+blood+rsfMRI	specific	0.647	2.777	0.007
		GMV+bh (spec)+blood	specific	0.656	3.045	0.004
		bh (neu)+rsfMRI	neutral	0.657	2.482	0.013
		GMV+bh (spec)+rsfMRI	specific	0.666	3.067	0.004
		GMV+bh (neu)+rsfMRI	neutral	0.667	2.614	0.01
		bh (neu)+blood+rsfMRI	neutral	0.668	3.207	0.003
		GMV+rsfMRI	intrinsic	0.673	2.966	0.005
		blood+rsfMRI	intrinsic	0.675	2.498	0.013
		GMV+bh (spec)+blood+rsfMRI	specific	0.675	2.543	0.012
		GMV+blood+rsfMRI	intrinsic	0.679	2.436	0.014
		GMV+bh (neu)+blood+rsfMRI	neutral	0.686	2.454	0.014
		GMV+bh (spec)	intrinsic	0.701	2.915	0.006
		bh (spec)+rsfMRI	specific	0.705	3.495	0.002
Patients vs Controls	None	GMV+bh (neu)	neutral	0.569	1.962	0.035
		GMV+bh (spec)	specific	0.59	2.548	0.012
		bh (spec)+blood+rsfMRI	specific	0.641	2.92	0.006
		GMV+bh (neu)+blood+rsfMRI	neutral	0.649	2.719	0.008
		blood+rsfMRI	intrinsic	0.652	3.256	0.003
		GMV+bh (spec)+blood+rsfMRI	specific	0.653	3.149	0.004
		GMV+blood+rsfMRI	intrinsic	0.653	3.207	0.003
		bh (neu)+blood+rsfMRI	specific	0.658	2.836	0.007

	bh (spec)+rsfMRI	specific	0.659	2.841	0.007
	GMV+rsfMRI	intrinsic	0.662	4.096	0.001
	GMV+bh (spec)+rsfMRI	specific	0.685	3.587	0.001
	GMV+bh (neu)+rsfMRI	neutral	0.694	4.143	0.001
	bh (neu)+rsfMRI	neutral	0.711	4.509	0.001

For each comparison, fold-wise scores were tested against chance level using a two-sided one-sample t-test; conf indicates any confounder included in the model (e.g., BMI). Stimuli specifies the feature subset used: spec = disorder-specific features, neu = neutral features, both = modalities without stimuli. Abbreviations: BN = bulimia nervosa; BED = binge-eating disorder; Patients = all clinical groups combined; Controls = healthy controls; owHC = overweight/obese healthy controls; nwHC = normal-weight healthy controls; BMI = body mass index; GMV = gray matter volume (structural MRI); rsfMRI = resting-state functional MRI; bh = behavioral measures; bh (spec) = behavioral task performance using food stimuli; bh (neu) = behavioral task performance using neutral stimuli; blood = peripheral blood biomarkers.

Regressions

Supplementary Table 10. Multimodal regression performance for binge-specific severity prediction.

target	groups	conf.	modality	stimuli	R2	*t*	*p*
Binge Specific	BED	BMI	bh (neu)+rsfMRI	neutral	0.185	1.926	.032
	BN	BMI	rsfMRI+task fMRI (neu)	neutral	0.131	1.942	.031

Only models that perform significantly better than chance are listed in the table. Abbreviations: BED = binge-eating disorder; BN = bulimia nervosa.

Supplementary Table 11. Multimodal regression performance for disease-unspecific severity prediction.

target	groups	conf.	modality	stimuli	R2	*t*	*p*
Disease Unspecific	BED	None	GMV+rsfMRI	intrinsic	0.202	2.862	.004
	BED	None	bh (neu)+rsfMRI	neutral	0.167	1.816	.040
	BED	None	GMV+bh (neu)+rsfMRI	neutral	0.117	1.833	.039
	BN	None	GMV+bh (neu)+rsfMRI	neutral	0.229	3.579	.001
	BN	None	GMV+rsfMRI	intrinsic	0.226	3.388	.001
	BN	None	bh (neu)+rsfMRI	neutral	0.218	3.131	.002
	HC	None	GMV+bh (spec)+rsfMRI	specific	0.217	2.739	.005
	HC	None	GMV+bh (neu)+rsfMRI	neutral	0.190	3.765	.000
	HC	None	GMV+bh (spec)+blood+rsfMRI	specific	0.184	2.507	.009
	HC	None	GMV+blood+rsfMRI	intrinsic	0.180	2.504	.009
	HC	None	bh (spec)+rsfMRI	specific	0.167	2.094	.023
	HC	None	bh (neu)+rsfMRI	neutral	0.167	3.270	.001
	HC	None	GMV+bh (neu)+blood+rsfMRI	neutral	0.162	2.232	.017
	HC	None	GMV+rsfMRI	intrinsic	0.139	2.518	.009
	PAT	None	bh (neu)+rsfMRI	specific	0.236	2.709	.006
	PAT	None	GMV+bh (neu)+rsfMRI	neutral	0.220	2.821	.004
	PAT	None	GMV+rsfMRI	intrinsic	0.220	4.765	.000
	all	None	GMV+rsfMRI	intrinsic	0.108	1.831	.039

Only multimodal models that perform significantly better than chance are listed in the table. Abbreviations: BED = binge-eating disorder; BN = bulimia nervosa. PAT = patients (all clinical groups combined); HC = healthy controls.

Supplementary Table 12. Multimodal regression performance for eating-specific severity prediction.

target	groups	conf.	modality	stimuli	R2	*t*	*p*
Eating Specific	HC	BMI	bh (spec)+rsfMRI	specific	0.266	2.264	.016
	HC	BMI	GMV+bh (spec)+rsfMRI	specific	0.263	2.301	.014
	HC	BMI	GMV+blood+rsfMRI	intrinsic	0.253	2.471	.010
	HC	BMI	GMV+bh (spec)+blood+rsfMRI	specific	0.252	2.423	.011
	HC	BMI	GMV+bh (neu)+blood+rsfMRI	neutral	0.239	2.462	.010
	HC	BMI	bh (neu)+rsfMRI	neutral	0.237	2.984	.003
	HC	BMI	bh (spec)+blood+rsfMRI	specific	0.236	2.012	.027
	HC	BMI	blood+rsfMRI	intrinsic	0.233	2.019	.026
	HC	BMI	bh (neu)+blood+rsfMRI	neutral	0.224	1.878	.035
	HC	BMI	GMV+bh (neu)+rsfMRI	neutral	0.223	3.742	.000
	HC	BMI	GMV+rsfMRI	intrinsic	0.202	4.357	.000

Only multimodal models that perform significantly better than chance are listed in the table. Abbreviations: BED = binge-eating disorder; BN = bulimia nervosa. PAT = patients (all clinical groups combined); HC = healthy controls.

Supplementary Table 13. Multimodal regression performance for eating-unspecific severity prediction.

target	groups	conf.	modality	stimuli	R2	*t*	*p*
Eating Unspecific	BED	BMI	bh (spec)+blood+rsfMRI	specific	0.187	1.904	.033
	BED	BMI	GMV+rsfMRI	intrinsic	0.185	2.184	.019
	BED	BMI	bh (neu)+rsfMRI	neutral	0.151	1.977	.029
	HC	BMI	GMV+rsfMRI	intrinsic	0.109	3.519	.001
	HC	BMI	GMV+bh (neu)+rsfMRI	neutral	0.104	1.967	.029
	PAT	BMI	bh (spec)+rsfMRI	specific	0.228	2.393	.012
	PAT	BMI	GMV+bh (spec)+rsfMRI	specific	0.190	1.920	.032
	PAT	BMI	bh (neu)+rsfMRI	neutral	0.180	2.929	.003
	PAT	BMI	GMV+rsfMRI	intrinsic	0.173	3.354	.001
	PAT	BMI	GMV+bh (neu)+rsfMRI	neutral	0.170	2.815	.004
	all	BMI	GMV+rsfMRI	intrinsic	0.108	2.473	.010

Only multimodal models that perform significantly better than chance are listed in the table. Abbreviations: BED= binge-eating disorder; BN = bulimia nervosa. PAT = patients (all clinical groups combined); HC = healthy controls.

Supplementary Table 14. Multimodal regression performance for weight history prediction.

target	groups	conf.	modality	stimuli	R2	*t*	*p*
Weight Fluctuations and Monitoring	BED	BMI	blood+rsfMRI	intrinsic	.249	2.540	0.008
	BED	BMI	bh (spec)+blood+rsfMRI	specific	.248	2.712	0.006
	BED	BMI	bh (spec)+rsfMRI	specific	.224	3.104	0.002
	BED	BMI	bh (neu)+blood+rsfMRI	neutral	.218	2.957	0.003
	BED	BMI	GMV+bh (spec)+blood+rsfMRI	specific	.184	2.714	0.006
	BED	BMI	GMV+bh (spec)+rsfMRI	specific	.178	3.102	0.002
	BED	BMI	GMV+rsfMRI	intrinsic	.167	2.859	0.004
	BED	BMI	GMV+blood+rsfMRI	specific	.164	2.309	0.014
	BED	BMI	GMV+bh (neu)+blood+rsfMRI	neutral	.158	2.383	0.012
	BED	BMI	bh (neu)+rsfMRI	neutral	.143	2.763	0.005
	BED	BMI	GMV+bh (neu)+rsfMRI	neutral	.114	2.475	0.010
	HC	BMI	GMV+bh (neu)+rsfMRI	neutral	.147	4.013	0.000
	HC	BMI	bh (neu)+rsfMRI	neutral	.138	3.436	0.001
	HC	BMI	GMV+bh (spec)+rsfMRI	specific	.131	2.568	0.008
	HC	BMI	GMV+rsfMRI	intrinsic	.119	3.261	0.001
	HC	BMI	GMV+bh (spec)+blood+rsfMRI	specific	.100	1.975	0.029
	HC	BMI	GMV+bh (neu)+blood+rsfMRI	neutral	.096	1.753	0.045

Only multimodal models that perform significantly better than chance are listed in the table. Bold rows represent multimodal models that performed significantly better than the best single modality for that group and target. Abbreviations: BED = binge-eating disorder; BN = bulimia nervosa. PAT = patients (all clinical groups combined); HC = healthy controls.

Supplementary Table 15. ML Regression Models that showed a significant gain compared to single Modality

Group	Target	best single R^2	Modalities	R^2	*P* welch	*P* paired
BED	Weight fluctuations	0.189	bh (spec)+blood+rsfMRI	0.248	0.056	0.038
BED			blood+rsfMRI	0.249	0.071	0.043
HC	Disease unspecific	0.119	GMV+bh (spec)+rsfMRI	0.212	0.001	0.000
HC			GMV+bh (neu)+rsfMRI	0.189	0.001	0.000
HC			bh (neu)+rsfMRI	0.167	0.021	0.000
HC			GMV+bh (spec)+blood+rsfMRI	0.171	0.042	0.000
HC			bh (spec)+rsfMRI	0.167	0.066	0.002
HC			GMV+blood+rsfMRI	0.166	0.071	0.001
HC			GMV+bh (neu)+blood+rsfMRI	0.151	0.198	0.016
HC	Weight fluctuations	0.121	GMV+bh (neu)+rsfMRI	0.146	0.094	0.011
HC			bh (neu)+rsfMRI	0.138	0.268	0.044

Values indicate mean R^2 across cross-validation folds. Best single model R^2 refers to the highest-performing unimodal reference model for the same target. Because samples for different modalities don’t completely overlap, we report both the paired model corrected t-test and Welch Test but interpret the paired t-test. Only significantly better models than the singe-modality reference are listed (paired t-test).
Abbreviations: bh=behavioral; neu=neutral task condition; spec=disorder-specific (food) task condition; rsfMRI=resting-state functional MRI; GMV=gray-matter volume; HC=healthy controls; BED=Binge-eating disorder.

References

1. Wittchen, H.-U., Zaudig, M. & Fydrich, T. SKID Strukturiertes Klinisches Interview für DSM-IV. Achse I und II. . *Zeitschrift für Klinische Psychologie und Psychotherapie* **28**, 68–70 (1997).
2. Grunert, S.C. Ein Inventar zur Erfassung von Selbstaussagen zum Ernährungsverhalten. [An inventory for determination of eating behaviors through self-reporting.]. *Diagnostica* **35**, 167–179 (1989).
3. Hilbert, A., Tuschen-Caffier, B. & Ohms, M. Eating Disorder Examination: Deutschsprachige Version des strukturierten Essstörungsinterviews. *Diagnostica* **50**, 98–106 (2004).
4. Beck, A.T., Steer, R.A. & Brown, G. (2011).
5. Preuss, U.W.*, et al.* [Psychometric evaluation of the German version of the Barratt Impulsiveness Scale]. *Nervenarzt* **79**, 305–319 (2008).
6. Nijs, I.M., Franken, I.H. & Muris, P. The modified Trait and State Food-Cravings Questionnaires: development and validation of a general index of food craving. *Appetite* **49**, 38–46 (2007).
7. Verbruggen, F., Logan, G.D. & Stevens, M.A. STOP-IT: Windows executable software for the stop-signal paradigm. *Behav Res Methods* **40**, 479–483 (2008).
8. Monsell, S. Task switching. *Trends Cogn Sci* **7**, 134–140 (2003).
9. Meiran, N. Reconfiguration of processing mode prior to task performance. *Journal of Experimental Psychology: Learning, Memory, and Cognition* **22**, 1423–1442 (1996).
10. Petermann, F. & Lepach, A.C. Wechsler memory scale. *in deutscher Übersetzung und Adaptation der WMS-IV von Davis Wechsler. Frankfurt a. M.: Pearson Assessment & Information GmbH* (2012).
11. Lehrl, S., Merz, J., Burkhard, G. & Fischer, B. *Mehrfach-wortschatz-intelligenztest MWT-B* (Spitta Verlag, Balingen, 2005).
12. Simon, J.J.*, et al.* Neural Food Reward Processing in Successful and Unsuccessful Weight Maintenance. *Obesity (Silver Spring)* **26**, 895–902 (2018).
13. Simon, J.J.*, et al.* Impaired Cross-Talk between Mesolimbic Food Reward Processing and Metabolic Signaling Predicts Body Mass Index. *Front Behav Neurosci* **8**, 359 (2014).
14. Simon, J.J.*, et al.* Neural signature of food reward processing in bulimic-type eating disorders. *Soc. Cogn. Affect. Neurosci.* **11**, 1393–1401 (2016).
15. Simon, J.J.*, et al.* Neural dissociation of food- and money-related reward processing using an abstract incentive delay task. *Soc Cogn Affect Neurosci* **10**, 1113–1120 (2015).
16. Simon, J.J.*, et al.* Integration of homeostatic signaling and food reward processing in the human brain. *JCI Insight* **2** (2017).
17. Skunde, M.*, et al.* Neural signature of behavioural inhibition in women with bulimia nervosa. *J Psychiatry Neurosci* **41**, E69–78 (2016).
18. Brett, M., Anton, J.-L., Valabregue, R. & Poline, J.-B. Region of interest analysis using an SPM toolbox. in *8th International Conferance on Functional Mapping of the Human Brain* (Sendai, Japan, 2002).
19. Rolls, E.T., Huang, C.C., Lin, C.P., Feng, J. & Joliot, M. Automated anatomical labelling atlas 3. *Neuroimage* **206**, 116189 (2020).

20. Nieto-Castanon, A. & Whitfield-Gabrieli, S. CONN Functional Connectivity Toolbox. RRID SCR_009550 (2025).
21. Nieto-Castanon, A. *Handbook of functional connectivity Magnetic Resonance Imaging methods in CONN* (2020).
22. Whitfield-Gabrieli, S., Nieto-Castanon, A. & Ghosh, S. Artifact detection tools (ART). *Cambridge, MA. Release Version* **7**, 11 (2011).
23. Power, J.D.*, et al.* Methods to detect, characterize, and remove motion artifact in resting state fMRI. *Neuroimage* **84**, 320–341 (2014).
24. Weis, S.*, et al.* Sex Classification by Resting State Brain Connectivity. *Cereb Cortex* **30**, 824–835 (2020).
25. Chen, H.J., Chuang, S.Y., Chang, H.Y. & Pan, W.H. Energy intake at different times of the day: Its association with elevated total and LDL cholesterol levels. *Nutr Metab Cardiovasc Dis* **29**, 390–397 (2019).
26. Eberle, E., Doering, A. & Keil, U. Weight change and change of total cholesterol and high-density-lipoprotein cholesterol. Results of the MONICA Augsburg cohort study. *Ann Epidemiol* **1**, 487–492 (1991).
27. Maher, T.*, et al.* Food Intake and Satiety Response after Medium-Chain Triglycerides Ingested as Solid or Liquid. *Nutrients* **11** (2019).
28. Suh, Y.J., Park, S.K., Choi, J.M. & Ryoo, J.H. The clinical importance of serum gamma-glutamyltransferase level as an early predictor of obesity development in Korean men. *Atherosclerosis* **227**, 437–441 (2013).
29. Vigil, P., Melendez, J., Petkovic, G. & Del Rio, J.P. The importance of estradiol for body weight regulation in women. *Front Endocrinol (Lausanne)* **13**, 951186 (2022).
30. Wyatt, P.*, et al.* Postprandial glycaemic dips predict appetite and energy intake in healthy individuals. *Nat Metab* **3**, 523–529 (2021).
31. Pedregosa, F.*, et al.* Scikit-learn: Machine learning in Python. *the Journal of machine Learning research* **12**, 2825–2830 (2011).
32. Bunnik, E.M.*, et al.* The mRNA-bound proteome of the human malaria parasite Plasmodium falciparum. *Genome Biol* **17**, 147 (2016).
