# Supplementary material for: Multimodal machine learning reveals neurobiological signatures of binge-type eating disorders
Source: Front Neurosci. 2026 Apr 13;20:1803154. doi: 10.3389/fnins.2026.1803154 (PMC13111282; doi:10.3389/fnins.2026.1803154)
Supplement: Supplementary file 3 [file Data_Sheet_3.pdf]

NeuroBED_ML: DATA EXPLORATION NOTEBOOK

This notebook performs data exploration and additional preprocessing of the NeuroBED dataset in preparation for machine learning using the `julearn` framework. At this point, the variables used for analyses have already been extracted from the raw data files and the data underwent minimal preprocessing (renaming variables, uniform naming convention for missing data etc.). Steps in this notebook include loading and cleaning data, handling missing values, removing outliers, and exporting structured outputs for modeling. The data will be written to file after each preprocessing step and stored here: `/02 - data_exploration/output/`.

The files used for analysis are additionally saved here: `/03 - data4ML`.

`allData_precleaned_step2.2c_Final.pkl`: All data
`data_cats.pkl`: Info about category-variable mapping.

Please note that some variables have been renamed in the paper for clarity:

fMRI = rsfMRI
MRI = GMV
CON_BED = owHC
CON_BN = nwHC
Weight history = weight fluctuations and monitoring

Table of Contents

- 0 Notebook Styling Params
- 1 Imports
  - 1.1 Variables
  - 1.2 Load Data
- 2 Preprocessing
  - 2.1 Data Categorization
    - 2.1.1 Full Categorization
    - 2.1.2 Model Categorization
      - 2.1.2.1 Features
      - 2.1.2.2 Targets
      - 2.1.2.2 Confounders & Others
  - 2.2 Outliers and Missing Data
  - 2.3 Save Preprocessed Data
- 4 Data Exploration
  - 4.1 Raw Data Inspection
  - 4.2 Final Sample Inspection
    - 4.2.1 Features
    - 4.2.2 Targets

Step	Description	Object(s)	Input File(s)	Output File(s)
1	Load input data (precleaned, selected variables)	`df_all`, `vars_all`, `rs_matrix`	`allData_precleaned.pkl`, `allVars_precleaned.pkl`, `NeuroBED_ML_AAL3Matrix_precleaned.pkl`	–
2	Convert all data columns to float, drop empty ones	`df_all`, `dropped_cols`, `colsb4`	–	`allData_precleaned_step1.pkl`, `allVars_precleaned_step1.pkl`
2.1a	Generate nested modality-feature mapping	`data_cats_full`	`NeuroBED_ML_Vars.xlsx`	–
2.1b	Generate final analysis categorization	`data_cats`	`data_cats_full`	-
2.1c	Add severity indices	`df_all`	–	`allData_precleaned_step2.1a.pkl`, `data_cats.pkl`, `data_cats_full.pkl`
2.2a	Calculate outliers/remove preselected subs	`zscore`, `zscore2`, `df_all2`, `allMissingData`	`df_all`	-
2.2b	Replace outlier data with NaN	`df_all3`, `rs_matrix`	`df_all2`, `allMissingData`	-
2.2c	Export Data and Cleaning summaries	`summary_df`, `allMissingData`, `df_all3`, `zscore`, `zscore2`, `rs_matrix`	–	`allData_precleaned_step2.2a_Z.pkl`, `allData_precleaned_step2.2b_Z.pkl`, `allData_precleaned_step2.2b.pkl`, `allData_precleaned_step2.2c_Final.pkl`, `subject_exclusion_summary.xlsx`, `data_cleaning_summary.csv`, `data_cleaning_summary.xlsx`, `AAL3Matrix_precleaned_step2.2c_Final.pkl`

0. NOTEBOOK STYLING PARAMS

In [1]:
```
# Printing style
from IPython.display import Markdown, display, Image,HTML
from pprint import pprint
def printmd(string):
    display(Markdown(string))
```

1. IMPORTS

In [2]:
```
import os, sys, random
from pathlib import Path

basepath = f"{os.getcwd()[:2]}/NeuroBed_ML/analysis/"

if not basepath in sys.path:
    sys.path.insert(0,basepath) # to get code from other files #"E:/NeuroBed_ML/analysis/"
    sys.path.insert(1, f"{basepath}05 - utils/") # to get code from other files
from NeuroBED_ML_helpers import *
from LR_Cmaps import *

import pandas as pd
import numpy as np
from scipy import stats
from scipy.stats import pearsonr
import matplotlib.pyplot as plt
from itertools import combinations
import matplotlib_inline
import seaborn as sns
import pickle,time
from datetime import datetime

# plotting parameters
try:
    plt.style.use(f'{basepath}05 - utils/LR_ST2.mplstyle')
    matplotlib_inline.backend_inline.set_matplotlib_formats('retina')
    fonts = ['Poppins','Montserrat','Manrope','Figtree','Helvetica','Roboto','Quicksand','Arial','AvenirLTPro-Book']
    plt.rcParams['font.family'] = fonts[random.randint(0,len(fonts)-1)]
except:
    plt.rcParams['font.family'] = 'Arial'
print ('current font: ', plt.rcParams['font.family'][0])
```

current font: Manrope

1.1 Variables

This section sets up the configuration, including naming conventions and I/O structure, and then loads the data:

- **Input Directory:** Points to extracted and minimally cleaned data under `/01 - data/`.
- **Output Directory:** Created if it doesn't exist. Located under `/02 - data_exploration/output/`.
- **Group Mapping:** Defines numerical IDs for each group:
  - `BED` → 1
  - `BN` → 2
  - `CON_BED (owHC)` → 3
  - `CON_BN (nwHC)` → 4

These group IDs are used for subject exclusion, stratified cross validation and plotting.

In [3]:
```
parent_dir   = basepath

in_dir       = resolve_path('data')
out_dir_ML   = resolve_path('data4ML') # for output files that are used for ML models. creates dir if it does not exist
out_dir_saf  = resolve_path('data_exploration/output/additional_data_safeties') # safety outputs. creates dir if it does not exist
out_dir_expl = resolve_path('data_exploration/output/data_cleaning') # exploration outputs. creates dir if it does not exist


groupNames     = {'BED':1,'CON_BED':3,'BN':2,'CON_BN':4}
groupNames_rev = { v: k for k, v in groupNames.items()}

saveFiles = False # save all output files from this notebook. Can be manually adjusted for each section
```

1.2 Load Data

The loaded dataset is already complete with all data types and pre-cleaned.

In [4]:
```
df_all    = pd.read_pickle(f"{in_dir}/NeuroBED_ML_allData_precleaned.pkl") # import all data (except rsfMRI)
rs_matrix = pd.read_pickle(f"{in_dir}/NeuroBED_ML_AAL3Matrix_precleaned.pkl") # import resting-state matrix (112x166x166)
vars_all  = pd.read_pickle(f"{in_dir}/NeuroBED_ML_allVars_precleaned.pkl") # variable info
```

2. PREPROCESSING

Before the features are categorized, empty features are removed from the dataset. The remaining feature values are converted to float for compatibility with the ML pipeline. The result is saved here: `allVars_precleaned_remEmptyCols.pkl/xlsx`

In [5]:
```
saveFile = saveFiles # use global parameter
```

In [6]:
```
# convert all data to float
df_all = df_all.apply(pd.to_numeric, errors='coerce')

# remove empty features. There are some MRI features where this happend
colsb4 = list(df_all.columns)
df_all.dropna(axis=1, how='all',inplace=True)
dropped_cols = list(set(colsb4) - set(list(df_all.columns)))
print ('dropped ',str(len(dropped_cols)), ' feature columns from the dataframe')
print (dropped_cols)
# remove these columns from the variable list
vars_all = vars_all[~vars_all['feature'].isin(dropped_cols)]
```

```
dropped  10  feature columns from the dataframe
['fMRI_MID_neu_Fmiddle_win_#_Cerebellum_10_L', 'fMRI_MID_neu_Fnull_#_Cerebellum_10_L', 'fMRI_MID_neu_Fhigh_loss_#_Cerebellum_10_L', 'fMRI_MID_neu_Fhighvsnull_#_Cerebellum_10_L', 'fMRI_MID_neu_Enull_#_Cerebellum
_10_L', 'fMRI_MID_neu_Ehighvsnull_#_Cerebellum_10_L', 'fMRI_MID_neu_Emiddle_#_Cerebellum_10_L', 'fMRI_MID_neu_Fmiddle_loss_#_Cerebellum_10_L', 'fMRI_MID_neu_Fhigh_win_#_Cerebellum_10_L', 'fMRI_MID_neu_Ehigh_#_C
erebellum_10_L']
```

2.1 Data Categorization

This section lists all variables (features), organized by modality and stimulus type, as defined in `data/variable_info/NeuroBED_ML_Vars.xlsx` (e.g., `category1 → category2 → category3`).

Because there are many categories with different numbers of variables, we also combine related items into clinically meaningful groups. These groups make the results easier to interpret and keep the analysis concise. We save these groupings so they can be reused when training machine-learning models with different sets of variables.

In [7]:
```
saveFile = saveFiles # use global parameter
```

2.1.1 Full Categorization

In [8]:
```
# Data categories and combinations
data_cats_full = {}
data_cats_full['cat1']      = vars_all.groupby(['category1'])['feature'].apply(list).to_dict()
data_cats_full['cat2']      = vars_all.groupby(['category2'])['feature'].apply(list).to_dict()
data_cats_full['cat3']      = vars_all.groupby(['category3'])['feature'].apply(list).to_dict()
data_cats_full['cat1cat2'] = vars_all.groupby(['category1','category2'])['feature'].apply(list).to_dict()
data_cats_full['cat1cat3'] = vars_all.groupby(['category1','category3'])['feature'].apply(list).to_dict()
data_cats_full['cat2cat3'] = vars_all.groupby(['category2','category3'])['feature'].apply(list).to_dict()
data_cats_full['cat1cat2cat3'] = vars_all.groupby(['category1','category2','category3'])['feature'].apply(list).to_dict()

if saveFile: # save this too for later
    with open(str(in_dir)+'data_cats_full.pkl', 'wb') as f:
        pickle.dump(data_cats_full, f)

vars_all.groupby(['category1','category2','category3']).describe()
# task fMRI: 136+2=138 (AAL3 minus thalamus + AAL2 thalamus)
# VBM: 166 (AAL3 only)
# rs: 165x166/2 = 13695 (AAL3 only minus diagonal)
```

Out[8]:

			feature			
			count	unique	top	freq
category1	category2	category3				
behavioral	CuedTask	all	4	4	cued_shift_rt	1
	GNG	neu	4	4	GNG_neu_gotrials_rt	1
		spec	4	4	GNG_spec_gotrials_rt	1
	MID	neu	3	3	MID_neu_high_reward	1
		spec	3	3	MID_spec_hight_reward	1
	RSS	all	4	4	RSS_target_rt	1
	StopSignal	all	3	3	sst_ssrt	1
	intelligence	all	1	1	mwtb	1
	working memory	all	2	2	wm_forward	1
blood	all	all	6	6	glucose	1
clinical	symptom	all	10	10	BMI	1
	weight_hist	all	11	11	hightest_weight	1
demographics	all	all	3	3	group	1
fMRI	GNG	neu	276	276	fMRI_GNG_neu_target_#_ACC_pre_L	1
		spec	276	276	fMRI_GNG_spec_notarget_#_ACC_pre_L	1

	MID	neu	1370	1370	fMRI_MID_neu_Ehighvsnull_#_ACC_pre_L	1
		spec	1380	1380	fMRI_MID_spec_Enone_#_ACC_pre_L	1
	RS	all	13695	13695	fMRI_RS_all_#_Precentral_L_to_Precentral_R	1
questionnaire	BDI	all	1	1	BDI	1
	BIS_BAS	all	5	5	BIS	1
	DEBQ	all	3	3	DEBQ_restrained_eating	1
	EDEQ	all	5	5	EDEQ_restraint	1
	FCQ	all	2	2	G_FCQ_state	1
sMRI	struct	all	166	166	sMRI_struct_all_VBM_#_ACC_pre_L	1

2.1.1 Model Categorization

This is the final grouping for the analyses.

In [9]:
```
data_cats = {'features': {},'targets': {},'confounders': {},'others': {}}
```

2.1.1.1 FEATURES

In [9]:
```
Image(filename='./plots/FeaturesOnly.png',width=300, height=150)
```

Out[9]:

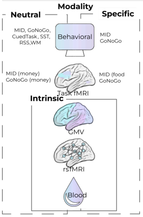

In [10]:
```
features =      {'bh_neu':        [('behavioral', 'CuedTask', 'all'),('behavioral', 'GNG', 'neu'),('behavioral', 'MID', 'neu'),('behavioral', 'RSS', 'all'),
                                           ('behavioral', 'StopSignal', 'all'),('behavioral', 'intelligence', 'all'),('behavioral', 'working memory', 'all')],
                 'bh_spec':       [('behavioral', 'GNG', 'spec'),('behavioral', 'MID', 'spec')],
                 'blood':         [('blood', 'all', 'all')],
                 'task_fMRI_spec':[('fMRI', 'GNG', 'spec'),('fMRI', 'MID', 'spec')],
                 'task_fMRI_neu': [('fMRI', 'GNG', 'neu'),('fMRI', 'MID', 'neu')],
                 'fMRI':          [('fMRI', 'RS', 'all')],
                 'MRI' :          [('sMRI', 'struct', 'all')]}

data_cats['confounders'] = ['sex','age','BMI']
data_cats['others']      = ['VAS_hungr_before','VAS_mood_before','VAS_hungr_after','VAS_mood after','hightest_weight',
                             'age_highest_weight','lowest_weight','age_lowest_weight','look','bodyshape_father','bodyshape_mother',
                            'first_time_overweight']


for mod,subkeys in features.items():
    data_cats['features'][mod] = [feat for tup in features[mod] for feat in data_cats_full['cat1cat2cat3'][tup]]

# Display the new category - feature mapping. Limit the display to x features
displayDictJupyter(data_cats['features'],val_lim=30)
```

Key	Value
bh_neu	['cued_shift_rt', 'cued_shift_err', 'cued_maintain_rt', 'cued_maintain_err', 'GNG_neu_gotrials_rt', 'GNG_neu_gotrials_err', 'GNG_neu_nogotrials_err', 'GNG_neu_nogotrials_rt', 'MID_neu_high_reward', 'MID_neu_middle_reward', 'MID_neu_no_reward', 'RSS_target_rt', 'RSS_nontarget_rt', 'RSS_target_err', 'RSS_nontarget_err', 'sst_ssrt', 'sst_ss_rt', 'sst_prs', 'mwtb', 'wm_forward', 'wm_backward']
bh_spec	['GNG_spec_gotrials_rt', 'GNG_spec_gotrials_err', 'GNG_spec_nogotrials_err', 'GNG_spec_nogotrials_rt', 'MID_spec_hight_reward', 'MID_spec_middle_reward', 'MID_spec_no_reward']
blood	['glucose', 'GGT', 'triglyceride', 'cholesterin', 'progesteron', 'oestradiol']
task_fMRI_spec	['fMRI_GNG_spec_notarget_#_ACC_pre_L', 'fMRI_GNG_spec_notarget_#_ACC_pre_R', 'fMRI_GNG_spec_notarget_#_ACC_sub_L', 'fMRI_GNG_spec_notarget_#_ACC_sub_R', 'fMRI_GNG_spec_notarget_#_ACC_sup_L', 'fMRI_GNG_spec_notarget_#_ACC_sup_R', 'fMRI_GNG_spec_notarget_#_Amygdala_L', 'fMRI_GNG_spec_notarget_#_Amygdala_R', 'fMRI_GNG_spec_notarget_#_Angular_L', 'fMRI_GNG_spec_notarget_#_Angular_R', 'fMRI_GNG_spec_notarget_#_Calcarine_L', 'fMRI_GNG_spec_notarget_#_Calcarine_R', 'fMRI_GNG_spec_notarget_#_Caudate_L', 'fMRI_GNG_spec_notarget_#_Caudate_R', 'fMRI_GNG_spec_notarget_#_Cerebellum_10_L', 'fMRI_GNG_spec_notarget_#_Cerebellum_10_R', 'fMRI_GNG_spec_notarget_#_Cerebellum_3_L', 'fMRI_GNG_spec_notarget_#_Cerebellum_3_R', 'fMRI_GNG_spec_notarget_#_Cerebellum_4_5_L', 'fMRI_GNG_spec_notarget_#_Cerebellum_4_5_R', 'fMRI_GNG_spec_notarget_#_Cerebellum_6_L', 'fMRI_GNG_spec_notarget_#_Cerebellum_6_R', 'fMRI_GNG_spec_notarget_#_Cerebellum_7b_L', 'fMRI_GNG_spec_notarget_#_Cerebellum_7b_R', 'fMRI_GNG_spec_notarget_#_Cerebellum_8_L', 'fMRI_GNG_spec_notarget_#_Cerebellum_8_R', 'fMRI_GNG_spec_notarget_#_Cerebellum_9_L', 'fMRI_GNG_spec_notarget_#_Cerebellum_9_R', 'fMRI_GNG_spec_notarget_#_Cerebellum_Crus1_L', 'fMRI_GNG_spec_notarget_#_Cerebellum_Crus1_R', '...']
task_fMRI_neu	['fMRI_GNG_neu_target_#_ACC_pre_L', 'fMRI_GNG_neu_target_#_ACC_pre_R', 'fMRI_GNG_neu_target_#_ACC_sub_L', 'fMRI_GNG_neu_target_#_ACC_sub_R', 'fMRI_GNG_neu_target_#_ACC_sup_L', 'fMRI_GNG_neu_target_#_ACC_sup_R', 'fMRI_GNG_neu_target_#_Amygdala_L', 'fMRI_GNG_neu_target_#_Amygdala_R', 'fMRI_GNG_neu_target_#_Angular_L', 'fMRI_GNG_neu_target_#_Angular_R', 'fMRI_GNG_neu_target_#_Calcarine_L', 'fMRI_GNG_neu_target_#_Calcarine_R', 'fMRI_GNG_neu_target_#_Caudate_L', 'fMRI_GNG_neu_target_#_Caudate_R', 'fMRI_GNG_neu_target_#_Cerebellum_10_L', 'fMRI_GNG_neu_target_#_Cerebellum_10_R', 'fMRI_GNG_neu_target_#_Cerebellum_3_L', 'fMRI_GNG_neu_target_#_Cerebellum_3_R', 'fMRI_GNG_neu_target_#_Cerebellum_4_5_L', 'fMRI_GNG_neu_target_#_Cerebellum_4_5_R', 'fMRI_GNG_neu_target_#_Cerebellum_6_L', 'fMRI_GNG_neu_target_#_Cerebellum_6_R', 'fMRI_GNG_neu_target_#_Cerebellum_7b_L', 'fMRI_GNG_neu_target_#_Cerebellum_7b_R', 'fMRI_GNG_neu_target_#_Cerebellum_8_L', 'fMRI_GNG_neu_target_#_Cerebellum_8_R', 'fMRI_GNG_neu_target_#_Cerebellum_9_L', 'fMRI_GNG_neu_target_#_Cerebellum_9_R', 'fMRI_GNG_neu_target_#_Cerebellum_Crus1_L', 'fMRI_GNG_neu_target_#_Cerebellum_Crus1_R', '...']
fMRI	['fMRI_RS_all_#_Precentral_L_to_Precentral_R', 'fMRI_RS_all_#_Precentral_L_to_Frontal_Sup_2_L', 'fMRI_RS_all_#_Precentral_R_to_Frontal_Sup_2_L', 'fMRI_RS_all_#_Precentral_L_to_Frontal_Sup_2_R', 'fMRI_RS_all_#_Precentral_R_to_Frontal_Sup_2_R', 'fMRI_RS_all_#_Frontal_Sup_2_L_to_Frontal_Sup_2_R', 'fMRI_RS_all_#_Precentral_L_to_Frontal_Mid_2_L', 'fMRI_RS_all_#_Precentral_R_to_Frontal_Mid_2_L', 'fMRI_RS_all_#_Frontal_Sup_2_L_to_Frontal_Mid_2_L', 'fMRI_RS_all_#_Frontal_Sup_2_R_to_Frontal_Mid_2_L', 'fMRI_RS_all_#_Precentral_L_to_Frontal_Mid_2_R', 'fMRI_RS_all_#_Precentral_R_to_Frontal_Mid_2_R', 'fMRI_RS_all_#_Frontal_Sup_2_L_to_Frontal_Mid_2_R', 'fMRI_RS_all_#_Frontal_Sup_2_R_to_Frontal_Mid_2_R', 'fMRI_RS_all_#_Frontal_Mid_2_L_to_Frontal_Mid_2_R', 'fMRI_RS_all_#_Precentral_L_to_Frontal_Inf_Oper_L', 'fMRI_RS_all_#_Precentral_R_to_Frontal_Inf_Oper_L', 'fMRI_RS_all_#_Frontal_Sup_2_L_to_Frontal_Inf_Oper_L', 'fMRI_RS_all_#_Frontal_Sup_2_R_to_Frontal_Inf_Oper_L', 'fMRI_RS_all_#_Frontal_Mid_2_L_to_Frontal_Inf_Oper_L', 'fMRI_RS_all_#_Frontal_Mid_2_R_to_Frontal_Inf_Oper_L', 'fMRI_RS_all_#_Precentral_L_to_Frontal_Inf_Oper_R', 'fMRI_RS_all_#_Precentral_R_to_Frontal_Inf_Oper_R', 'fMRI_RS_all_#_Frontal_Sup_2_L_to_Frontal_Inf_Oper_R', 'fMRI_RS_all_#_Frontal_Sup_2_R_to_Frontal_Inf_Oper_R', 'fMRI_RS_all_#_Frontal_Mid_2_L_to_Frontal_Inf_Oper_R', 'fMRI_RS_all_#_Frontal_Mid_2_R_to_Frontal_Inf_Oper_R', 'fMRI_RS_all_#_Frontal_Inf_Oper_L_to_Frontal_Inf_Oper_R', 'fMRI_RS_all_#_Precentral_L_to_Frontal_Inf_Tri_L', 'fMRI_RS_all_#_Precentral_R_to_Frontal_Inf_Tri_L', '...']
MRI	['sMRI_struct_all_VBM_#_ACC_pre_L', 'sMRI_struct_all_VBM_#_ACC_pre_R', 'sMRI_struct_all_VBM_#_ACC_sub_L', 'sMRI_struct_all_VBM_#_ACC_sub_R', 'sMRI_struct_all_VBM_#_ACC_sup_L', 'sMRI_struct_all_VBM_#_ACC_sup_R', 'sMRI_struct_all_VBM_#_Amygdala_L', 'sMRI_struct_all_VBM_#_Amygdala_R', 'sMRI_struct_all_VBM_#_Angular_L', 'sMRI_struct_all_VBM_#_Angular_R', 'sMRI_struct_all_VBM_#_Calcarine_L', 'sMRI_struct_all_VBM_#_Calcarine_R', 'sMRI_struct_all_VBM_#_Caudate_L', 'sMRI_struct_all_VBM_#_Caudate_R', 'sMRI_struct_all_VBM_#_Cerebellum_10_L', 'sMRI_struct_all_VBM_#_Cerebellum_10_R', 'sMRI_struct_all_VBM_#_Cerebellum_3_L', 'sMRI_struct_all_VBM_#_Cerebellum_3_R', 'sMRI_struct_all_VBM_#_Cerebellum_4_5_L', 'sMRI_struct_all_VBM_#_Cerebellum_4_5_R', 'sMRI_struct_all_VBM_#_Cerebellum_6_L', 'sMRI_struct_all_VBM_#_Cerebellum_6_R', 'sMRI_struct_all_VBM_#_Cerebellum_7b_L', 'sMRI_struct_all_VBM_#_Cerebellum_7b_R', 'sMRI_struct_all_VBM_#_Cerebellum_8_L', 'sMRI_struct_all_VBM_#_Cerebellum_8_R', 'sMRI_struct_all_VBM_#_Cerebellum_9_L', 'sMRI_struct_all_VBM_#_Cerebellum_9_R', 'sMRI_struct_all_VBM_#_Cerebellum_Crus1_L', 'sMRI_struct_all_VBM_#_Cerebellum_Crus1_R', '...']

2.1.1.2 TARGETS

To measure how severe the symptoms were across different areas, we created summary scores (composite indices) from selected questionnaire features. These scores are based on values that were first standardized to a common scale (ranging from 0 to 1). In some cases, features were given more or less weight to reflect their clinical importance within a domain.

We evaluated five symptom areas:

1. **`Disease unspecific`** – general depressive symptoms (e.g., BDI)
2. **`Weight history`** – history of weight changes and frequency of self-weighing
3. **`Eating unspecific`** – general eating behaviors (DEBQ and FCQ subscales)
4. **`Eating specific`** – eating disorder–related symptoms (EDEQ total score)
5. **`Binge specific`** – frequency of binge eating episodes

The severity scores were calculated in two steps:

- Each feature was rescaled to a 0–1 range so that all measures were comparable.
- The features were then averaged, either equally or with weights applied, to produce one score per symptom area.

For the **Eating unspecific** area, we weighted the features so that the DEBQ (3 items) and the FCQ (2 items) each contributed equally (50% each). A similar approach was used for the **Weight history** area, with equal weight (50% each) given to weight fluctuations and number of weigh-ins.

The final scores were added to the dataset with the suffix `_severity` (e.g., `Disease_unspecific_severity`).

In [11]:
```
severity_idx = {'Disease_unspecific': ['BDI'],
                'Weight_history':     ['nr_gains_plus5','nr_gains_plus6to10','nr_gains_plus11to20','nr_gains_plus21to30','nr_gains_plus31to40',
                                       'nr_gains_plus40','n_weighings'],
                'Eating_unspecific':  ['DEBQ_restrained_eating', 'DEBQ_emotional_eating', 'DEBQ_external_eating','G_FCQ_state', 'G_FCQ_trait'],
                'Eating_specific':    ['EDEQ_total_score'],
                'Binge_specific':     ['n_binges'],
               }

weights    = {'Eating_unspecific': 3*[0.5/3] + 2*[0.25], # weights of the individual factors to the total score (e.g. 50% DEBQ + 50% FCQ)
              'Weight_history':    6*[0.5/6] + [0.55]}

# calculate the severity indices
suffix = '_severity'
df_all = compute_weighted_severity_indices(df_all, severity_idx, weights,suffix=suffix)

# Assign the new severity indices names to the data_cats dict
data_cats['targets'] = {f"{key}{suffix}": value for key, value in severity_idx.items()}
```

2.1.1.3 CONFOUNDERS & OTHERS

In [12]:
```
data_cats['confounders'] = ['sex','age','BMI']
data_cats['others']      = ['VAS_hungr_before','VAS_mood_before','VAS_hungr_after','VAS_mood after','hightest_weight',
                            'age_highest_weight','lowest_weight','age_lowest_weight','look','bodyshape_father','bodyshape_mother',
                           'first_time_overweight']
```

2.2 Outliers and Missing Data

To maintain data quality across different types of measures, we flagged subjects with too much missing or unusable information. This included both missing values and extreme outliers (based on z-scores) across categories such as behavioral, blood, clinical, demographics, fMRI, questionnaires, and sMRI.

Subjects were flagged for exclusion if they met either of these thresholds:

- **Missing data cutoff**: more than 50% missing in a category
- **Z-score threshold**: values above 10 (extreme outliers)

In addition, 9 subjects were removed based on prior knowledge or manual review: `K020SJ`, `K030CN`, `K031KB`, `K089KP`, `K091AH`, `P010SV`, `P011GR`, `P022KB`, `P025JB`.

A summary matrix (`data_cleaning_summary`) was created to track missing and flagged values for each subject and category. Exclusion was decided separately for each category and flagged if the proportion of missing data or outliers exceeded the set thresholds.

Instead of removing entire subjects, only the flagged categories were set to `NaN`. This allowed subjects with partial data to still contribute to analyses in other categories.

The following output files were saved:

- `data_cleaning_summary.csv` / `.xlsx` – summary of missing values and outliers by subject and category
- `subject_exclusion_summary.xlsx` – simplified version of the cleaning summary showing excluded subjects by category
- `allData_precleaned_step2.2a_Z.pkl` – z-scored version of the main dataframe
- `allData_precleaned_step2.2b_Z.pkl` – z-scored dataframe with preselected subjects removed
- `allData_precleaned_step2.2b.pkl` – main dataframe with preselected subjects removed
- `allData_precleaned_step2.2c_Final.pkl` – final dataframe with outlier data removed

(a) calculate outliers + remove preselected subjects

In [13]:
```
                  # features: multiple columns for each modality. Can take some missing data
missing_cutoff = {'bh_neu': 0.5,'bh_spec': 0.5, 'blood': 0.5,'task_fMRI_spec': 0.5,'task_fMRI_neu': 0.5,'fMRI': 0.5, 'MRI': 0.5,
                  # targets: only one column. so if the value is missing, the subject will be excluded for that regression analysis.
                  'Disease_unspecific_severity': 0.5, 'Weight_history_severity': 0.5,'Eating_unspecific_severity': 0.5,
                  'Eating_specific_severity': 0.5,'Binge_specific_severity':0.5}
                  # features
zthresholds =    {'bh_neu': 10,'bh_spec': 10, 'blood': 10,'task_fMRI_spec': 10,'task_fMRI_neu': 10,'fMRI': 10, 'MRI': 10,
                  # targets: Dont exclude anybody based on z scores here
                  'Disease_unspecific_severity': None, 'Weight_history_severity': None,'Eating_unspecific_severity': None,
                  'Eating_specific_severity': None,'Binge_specific_severity':None}

subs2rem       = ['K020SJ','K030CN','K031KB','K089KP','K091AH','P010SV','P011GR','P022KB','P025JB']

# create a z scored version of that dataframe. Use only float columns
zscore = pd.DataFrame(np.abs(stats.zscore(df_all.select_dtypes(include=["float"]),nan_policy='omit')),columns=df_all.columns,index=df_all.index)


# Remove pre-selected subjects from both zscore and df_all
zscore2           = zscore.drop(index=subs2rem, errors='ignore')
df_all2           = df_all.drop(index=subs2rem, errors='ignore')
allMissingData    = pd.DataFrame(index = df_all2.index) # collect all information here
missing_data_list = [] # Prepare a list to collect the missing data for each category combination

# We check missing and outlier data only for features and targets
for modality, features in getCatDataMapping(my_dict=data_cats).items():

    # Pre-compute thresholds to avoid repetitive dictionary lookups
    z_thresh = zthresholds[modality]
    missing_thresh = missing_cutoff[modality]

    # Vectorized calculations for the missing data and exclusion flags
    if z_thresh is not None:
        zscore_above_thresh = zscore2[features].gt(z_thresh).sum(axis=1)
    else: # Don't apply any z-threshold filtering
        zscore_above_thresh = pd.Series(0, index=zscore2.index)

    missing_features    = zscore2[features].isnull().sum(axis=1)
    total_features      = len(features)
    missing_total       = (zscore_above_thresh + missing_features) / total_features
    exclusion_flag      = (missing_total > missing_thresh)

    # Create a DataFrame for the current category and append to the list
    missing_data = pd.DataFrame({
        f'zthresh_{z_thresh}_{modality}': zscore_above_thresh,
        f'missing_{modality}': missing_features,
        f'ntotal_{modality}': total_features,
        f'missingtotal_{modality}': missing_total,
        f'exclusion_{modality}': exclusion_flag
    })

    missing_data_list.append(missing_data)

# Concatenate all the missing data into a single DataFrame
allMissingData = pd.concat(missing_data_list, axis=1)
```

(b) replace outlier data with NaNs

In [14]:
```
# remove subject data that was flagged for exclusion (we do this individually for each category so subjects may remain in the dataframe)
df_all3 = df_all2.copy() # removed subject data will be replaced with NaN and then excluded when we split the frame

for modality, features in getCatDataMapping(my_dict=data_cats).items():

    missing_thresh = missing_cutoff[modality]
    curr_cols      = allMissingData.filter(like = 'exclusion_'+modality) # list of flagged subjects

    if len(curr_cols) > 1: # if we have more than one exclusion flag per category, take the mean
        curr_flag = curr_cols.mean(axis=1) > missing_thresh
    else:
        curr_flag = curr_cols
    printmd('**' +modality+':** ' + str(len(curr_flag.loc[curr_flag==True])) +' subjects were excluded') # number of subjects to be exclusion
    printmd(str(curr_flag.loc[curr_flag==True])) # number of subjects to be exclusion
    printmd(str(len(curr_flag.loc[curr_flag==False])) +' subjects were used') # number of subjects to be exclusion
```

```
    # mark all flagged subject data as nan. This is done per datatype because subjects may have enough good data
    # of some datatypes and not enough of the others. So only the data of bad datatypes are removed but the subject stays in the analysis for now
    for val in features:
        df_all3[val]=np.where(curr_flag==True, np.nan, df_all3[val])

    # For resting-state data, do the same with the full matrix used for analyses
    if modality == 'fMRI':
        flagged_subs = curr_flag.loc[curr_flag==True].index.tolist() # if there is any data for these subjects, set it to NaN
        for key in flagged_subs:
            if key in rs_matrix:
                rs_matrix[key][:] = np.nan  # Sets the existing array's values to NaN


for group_name, sub_df in df_all3.groupby('group'):
    print(f"\nGroup: {groupNames_rev[group_name]}")
    print(f"Count: {len(sub_df)}")
    print("Subjects (index):")
    print(sub_df.index.tolist())
    print ('-----')
```

bh_neu: 0 subjects were excluded

Series([], dtype: bool)

110 subjects were used

bh_spec: 3 subjects were excluded

subjects P103MS True P109AN True K110NH True dtype: bool

107 subjects were used

blood: 3 subjects were excluded

subjects P003MS True K105HK True K003AL True dtype: bool

107 subjects were used

task_fMRI_spec: 2 subjects were excluded

subjects P006AK True P103MS True dtype: bool

108 subjects were used

task_fMRI_neu: 2 subjects were excluded

subjects P006AK True P103MS True dtype: bool

108 subjects were used

fMRI: 2 subjects were excluded

subjects P006AK True P103MS True dtype: bool

108 subjects were used

MRI: 1 subjects were excluded

subjects P006AK True dtype: bool

109 subjects were used

Disease_unspecific_severity: 0 subjects were excluded

Series([], dtype: bool)

110 subjects were used

Weight_history_severity: 0 subjects were excluded

Series([], dtype: bool)

110 subjects were used

Eating_unspecific_severity: 0 subjects were excluded

Series([], dtype: bool)

110 subjects were used

Eating_specific_severity: 1 subjects were excluded

subjects P007AS True dtype: bool

109 subjects were used

Binge_specific_severity: 55 subjects were excluded

subjects K081RB True K082AS True K083RW True K084BH True K085MG True K086WP True K087RW True K088CM True K090LA True K092ER True K093TK True K094YS True K095KZ True K096PS True K097LM True K098EW True K099MP True K100HA True K101PF True K102NS True K103LK True K104EG True K105HK True K106TK True K107VK True K108JF True K109MG True K110NH True K001DO True K002UW True K003AL True K004KH True K005CH True K006JR True K007AT True K008JS True K009AU True K010SH True K011CU True K012TN True K014KW True K015CM True K016MB True K017LS True K018PS True K019AL True K021RM True K022LZ True K023TS True K024KK True K025JJ True K026MM True K027SH True K028NW True K029NK True dtype: bool

55 subjects were used

```
Group: BED
Count: 26
Subjects (index):
['P001AH', 'P002KB', 'P003MS', 'P004US', 'P005MF', 'P006AK', 'P007AS', 'P008NB', 'P009PH', 'P012KR', 'P013MP', 'P014SS', 'P015AL', 'P016OH', 'P017GV', 'P018EV', 'P019ME', 'P020JE', 'P021AS', 'P023MW', 'P024VC',
'P026ST', 'P027IG', 'P028SP', 'P029AR', 'P030KM']
-----

Group: BN
Count: 29
Subjects (index):
['P081LH', 'P082AS', 'P083LG', 'P084RS', 'P085SF', 'P086SS', 'P087HS', 'P088JF', 'P089GE', 'P090RG', 'P091GH', 'P092JG', 'P093BG', 'P094CC', 'P095MS', 'P096TB', 'P097MW', 'P098ST', 'P099CW', 'P100DK', 'P101SD',
'P102EB', 'P103MS', 'P104PN', 'P105ML', 'P106UP', 'P107JK', 'P108AM', 'P109AN']
-----

Group: CON_BED
Count: 28
Subjects (index):
['K081RB', 'K082AS', 'K083RW', 'K084BH', 'K085MG', 'K086WP', 'K087RW', 'K088CM', 'K090LA', 'K092ER', 'K093TK', 'K094YS', 'K095KZ', 'K096PS', 'K097LM', 'K098EW', 'K099MP', 'K100HA', 'K101PF', 'K102NS', 'K103LK',
'K104EG', 'K105HK', 'K106TK', 'K107VK', 'K108JF', 'K109MG', 'K110NH']
-----

Group: CON_BN
Count: 27
Subjects (index):
['K001DO', 'K002UW', 'K003AL', 'K004KH', 'K005CH', 'K006JR', 'K007AT', 'K008JS', 'K009AU', 'K010SH', 'K011CU', 'K012TN', 'K014KW', 'K015CM', 'K016MB', 'K017LS', 'K018PS', 'K019AL', 'K021RM', 'K022LZ', 'K023TS',
'K024KK', 'K025JJ', 'K026MM', 'K027SH', 'K028NW', 'K029NK']
-----
```

c) manual data exclusion

In [15]:
```
df_all3.loc[df_all3['glucose'] < 60, 'glucose'] = np.nan # exclude glucose values below 60 (4 subjects)
df_all3.loc[df_all3['triglyceride'] < 10, ['cholesterin','triglyceride']] = np.nan # exclude trygliceride values below 10 (3 subjects). Remove the same subjects for extremely low cholesterine values
df_all3.loc['K098EW', data_cats['features']['blood']] = np.nan # this subjects has more than half of the blood values missing now, so we remove his blood data completely

# check individual subjects
#df_all3.loc['P104PN', data_cats['features']['blood']]

#df_all3.groupby(['group','sex'])['sex'].describe()
```

2.3 Save Preprocessed Data

In [15]:
```
saveFile = False
```

In [16]:
```
if saveFile:
    print ('saving preprocessed files...')

    # create an overview over the exclusions
    # Start with subject index and group
    summary_df = df_all[['group']].copy()
    summary_df = summary_df.loc[summary_df.index.union(subs2rem)]  # Ensure pre-excluded are included

    # Add group name
    summary_df['group_name'] = summary_df['group'].map({v: k for k, v in groupNames.items()})

    # Add pre-exclusion flag
    summary_df['pre_excluded'] = summary_df.index.isin(subs2rem)

    # Add all exclusion flags from the main missing data check
    exclusion_cols = [col for col in allMissingData.columns if col.startswith('exclusion_')]
    summary_df = summary_df.join(allMissingData[exclusion_cols], how='left')
```

```python
    # Fill NA exclusion flags with False for subjects not evaluated (e.g., pre-excluded)
    pd.set_option('future.no_silent_downcasting', True)
    summary_df[exclusion_cols] = summary_df[exclusion_cols].apply(lambda col: col.fillna(False).astype(bool))

    # (1) data for ML analyses
    df_all3.drop(columns=df_all3.filter(like='_RS_').columns).to_pickle(f'{out_dir_ML}/allData_precleaned_step2.2c_Final.pkl') # with outliers replaced by nans + rs columns dropped
    with open(f'{out_dir_ML}/data_cats.pkl', 'wb') as f:
        pickle.dump(data_cats, f)
    with open(f'{out_dir_ML}/AAL3Matrix_precleaned_step2.2c_Final.pkl', 'wb') as f:
        pickle.dump(rs_matrix, f)

    # (2) additional data safeties
    allMissingData.to_csv(f'{out_dir_expl}/data_cleaning_summary.csv', index=True)
    allMissingData.to_excel(f'{out_dir_expl}/data_cleaning_summary.xlsx', index=True)
    summary_df.to_excel(f'{out_dir_expl}/subject_exclusion_summary.xlsx', index=True)
    vars_all.to_pickle(f'{out_dir_saf}/allVars_precleaned_step1.pkl') # variable list without empty columns
    zscore.to_pickle(f'{out_dir_saf}/allData_precleaned_step2.2a_Z.pkl') # zscored version of df_all
    zscore2.to_pickle(f'{out_dir_saf}/allData_precleaned_step2.2b_Z.pkl') # with pre-selected subs removed
    df_all.to_pickle(f'{out_dir_saf}/allData_precleaned_step1.pkl') # data without empty columns
    df_all.to_pickle(f'{out_dir_saf}/allData_precleaned_step2.1a.pkl') #  added severity indices
    df_all2.to_pickle(f'{out_dir_saf}/allData_precleaned_step2.2b.pkl') # with pre-selected subs removed
    df_all3.to_pickle(f'{out_dir_saf}/allData_precleaned_step2.2c_Final.pkl') # with outliers replaced by nans

    # save both data categorizations
    with open(f'{out_dir_saf}/data_cats.pkl', 'wb') as f:
        pickle.dump(data_cats, f)
    with open(f'{out_dir_saf}/data_cats_full.pkl', 'wb') as f:
        pickle.dump(data_cats_full, f)
    print ('Done!')
else:
    print ('Not saving files due to saveFile=False')
```

```
Not saving files due to saveFile=False
```

3 DATA EXPLORATION

3.1 Raw Data Inspection

For each modality, outliers were examined independently, regardless of how the data were grouped for later analysis. Outliers were also checked visually. In the behavioral data, outliers in error scores appeared as sharp peaks, usually when subjects made no errors.

In [26]:
```python
saveFile    = True#saveFiles # use global parameter
z_score_out = Path(out_dir_expl).parent.joinpath('z_thresholds')
z_score_out.mkdir(exist_ok=True)
```

In [27]:
```python
NonMRICats = [i for i in data_cats_full['cat1'] if 'MRI' not in i]

for cat in NonMRICats[:]:
    curr_cols = vars_all.loc[vars_all['category1']==cat]['feature'].tolist()
    curr_data = df_all2[curr_cols]
    #print (curr_data.describe())
    printmd("**----------------------------- "+cat+" ------------------------------**")

    if saveFile:
        zscored = convert_display_zScore(curr_data,zthresh=3,saveStats=str(z_score_out)+'/BH_threshs'+cat+'.csv')
    else:
        zscored = convert_display_zScore(curr_data,zthresh=3,saveStats=None)
```

----------------------------- **behavioral** -----------------------------

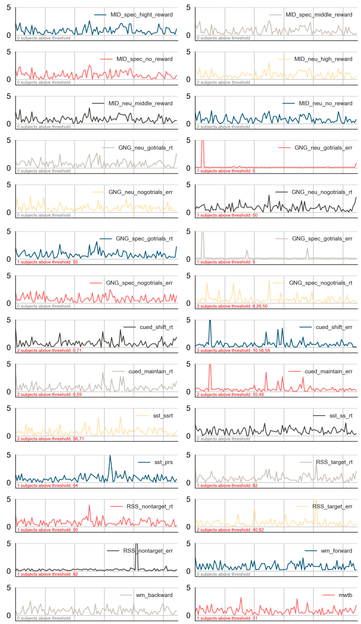

----------------------------- **blood** -----------------------------

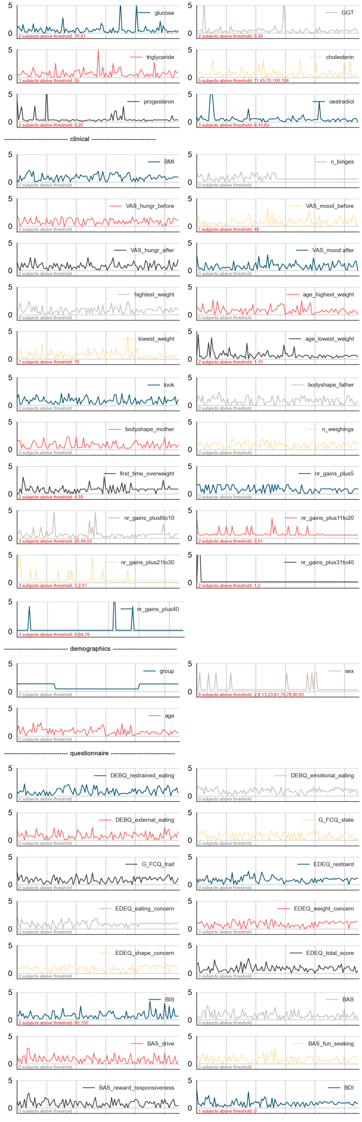

glucose
2 subjects above threshold: 70,81
GGT
2 subjects above threshold: 5,59
triglyceride
1 subjects above threshold: 55
cholesterin
5 subjects above threshold: 11,45,70,100,104
progesteron
2 subjects above threshold: 0,20
oestradiol
3 subjects above threshold: 9,10,83
clinical
BMI
0 subjects above threshold
n_binges
0 subjects above threshold
VAS_hungr_before
0 subjects above threshold
VAS_mood_before
1 subjects above threshold: 48
VAS_hungr_after
0 subjects above threshold
VAS_mood after
0 subjects above threshold
hightest_weight
0 subjects above threshold
age_highest_weight
0 subjects above threshold
lowest_weight
1 subjects above threshold: 75
age_lowest_weight
2 subjects above threshold: 1,10
look
0 subjects above threshold
bodyshape_father
0 subjects above threshold
bodyshape_mother
0 subjects above threshold
n_weighings
0 subjects above threshold
first_time_overweight
2 subjects above threshold: 4,59
nr_gains_plus5
0 subjects above threshold
nr_gains_plus6to10
3 subjects above threshold: 25,49,53
nr_gains_plus11to20
2 subjects above threshold: 0,51
nr_gains_plus21to30
3 subjects above threshold: 1,2,51
nr_gains_plus31to40
2 subjects above threshold: 1,2
nr_gains_plus40
3 subjects above threshold: 8,64,76
demographics
group
0 subjects above threshold
sex
9 subjects above threshold: 2,8,13,23,61,75,78,80,81
age
0 subjects above threshold
questionnaire
DEBQ_restrained_eating
0 subjects above threshold
DEBQ_emotional_eating
0 subjects above threshold
DEBQ_external_eating
0 subjects above threshold
G_FCQ_state
0 subjects above threshold
G_FCQ_trait
0 subjects above threshold
EDEQ_restraint
0 subjects above threshold
EDEQ_eating_concern
0 subjects above threshold
EDEQ_weight_concern
0 subjects above threshold
EDEQ_shape_concern
0 subjects above threshold
EDEQ_total_score
0 subjects above threshold
BIS
2 subjects above threshold: 90,100
BAS
0 subjects above threshold
BAS_drive
0 subjects above threshold
BAS_fun_seeking
0 subjects above threshold
BAS_reward_responsiveness
0 subjects above threshold
BDI
1 subjects above threshold: 0

```
In [28]: curr_cols = vars_all.loc[vars_all['category1']=='sMRI']['feature'].tolist()
curr_data = df_all[curr_cols]
printmd("**------------------------------ VBM ------------------------------**")
#print (curr_data.describe()[:5])
if saveFile:
    zscored = convert_display_zScore(curr_data.iloc[:, :20],zthresh=3,saveStats=str(z_score_out)+'/VBM_threshs.csv')
else:
    zscored = convert_display_zScore(curr_data.iloc[:, :20],zthresh=3,saveStats=None)
```

------------------------------ **VBM** ------------------------------

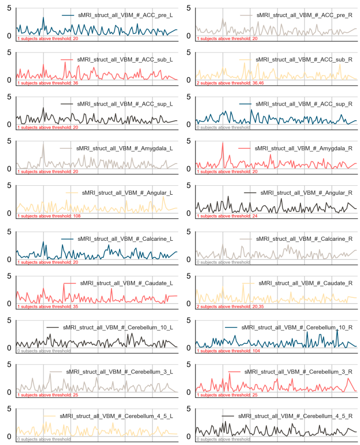


```
In [29]: TaskfMRICats = vars_all.loc[(vars_all['category1']=='fMRI')&(vars_all['category2'].isin(['MID', 'GNG']))]['category2'].unique()


for cat in TaskfMRICats:
    curr_cols = vars_all.loc[(vars_all['category1']=='fMRI')&(vars_all['category2'].isin([cat]))]['feature'].tolist()
    curr_data = df_all[curr_cols]
    printmd("**------------------------------ "+cat+" ------------------------------**")
    #print (curr_data.describe())
    if saveFile:
        zscored = convert_display_zScore(curr_data.iloc[:, :20],zthresh=3,saveStats=str(z_score_out)+'/TaskfMRI_threshs'+cat+'.csv')
    else:
        zscored = convert_display_zScore(curr_data.iloc[:, :20],zthresh=3,saveStats=None)
```

------------------------------ **GNG** ------------------------------

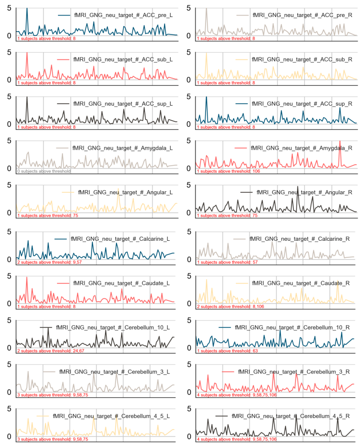

------------------------------ **MID** ------------------------------

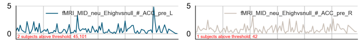

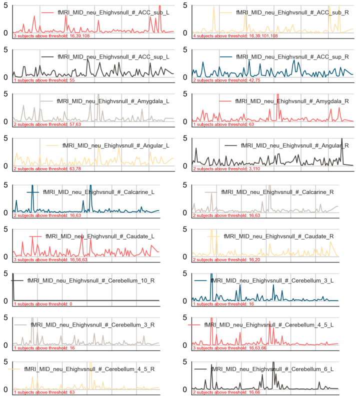

In [30]:
```python
curr_cols = vars_all.loc[vars_all['category2']=='RS']['feature'].tolist()
curr_data = df_all[curr_cols]
printmd("**------------------------------ Resting-State functional connectivity ------------------------------**")
#print (curr_data.describe()[:5])
if saveFile:
    zscored = convert_display_zScore(curr_data.iloc[:, :10],zthresh=3,saveStats=str(z_score_out)+'/RS_threshs.csv')
else:
    zscored = convert_display_zScore(curr_data.iloc[:, :10],zthresh=3,saveStats=None)
```

------------------------------ Resting-State functional connectivity ------------------------------

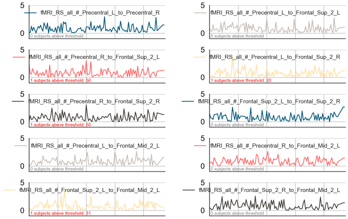

3.2 Final Sample Inspection

3.2.1 Features

We check the intercorrelation between the features of each modality for the entire sample. The code can be easily adjusted to check just for specific groups or across modalities

In [19]:
```python
saveFile = False#saveFiles # use global parameter
corr_out = Path(out_dir_expl).parent.joinpath('correlations')
corr_out.mkdir(exist_ok=True)
```

(a) Correlations

In [20]:
```python
group_combs    = {'BED':[1.0],'CON_BED':[3.0],'BN':[2.0],'CON_BN':[4.0],'PAT':[1.0,2.0],
                  'HC':[3.0,4.0],'O_weight':[1.0,3.0],'N_weight':[2.0,4.0],'All':[1.0,2.0,3.0,4.0]}

group_combs    = {'All':[1.0,2.0,3.0,4.0]} # do all just for now

for curr_feat_name,curr_cols in data_cats['features'].items():
    if curr_feat_name == 'fMRI': # no need to run this for RS data because this is already a correlation matrix
        continue
    print (curr_feat_name)
    for group_name,group_idx in group_combs.items():
        print (group_name)
        curr_data = df_all3.loc[df_all3['group'].isin(group_idx)][curr_cols].dropna(axis = 0, how = 'all')

        # We save these as a file, so we dont have to run it again. We can plot it with the fromFile key
        # dont show table if the category has many features
            #printmd("**------------------------------" + curr_feat_name + " features Correlation Matrix ------------------------------**")
        showtable = False

        rho,pval,ax = corrMatrix(curr_data,table=showtable,plot=False,cmap="RdBu_r",
                                 saveFile=f'{str(corr_out)}/{curr_feat_name}_{group_name}')
```

```
bh_neu
All
Found existing correlation matrices; loading from disk: D:\NeuroBed_ML\analysis\02 - data_exploration\output\correlations/bh_neu_All
bh_spec
All
Found existing correlation matrices; loading from disk: D:\NeuroBed_ML\analysis\02 - data_exploration\output\correlations/bh_spec_All
blood
All
Found existing correlation matrices; loading from disk: D:\NeuroBed_ML\analysis\02 - data_exploration\output\correlations/blood_All
task_fMRI_spec
All
Found existing correlation matrices; loading from disk: D:\NeuroBed_ML\analysis\02 - data_exploration\output\correlations/task_fMRI_spec_All
task_fMRI_neu
All
Found existing correlation matrices; loading from disk: D:\NeuroBed_ML\analysis\02 - data_exploration\output\correlations/task_fMRI_neu_All
MRI
All
Found existing correlation matrices; loading from disk: D:\NeuroBed_ML\analysis\02 - data_exploration\output\correlations/MRI_All
```

Here we check the level of significant corrations within feature modalities.

In [21]:
```python
for curr_mod_name,curr_cols in data_cats['features'].items():
    curr_data = df_all3[curr_cols].dropna(axis = 0, how = 'all')

    printmd(f"**------------------------------ {curr_mod_name} features Intercorrelations ------------------------------**")

    # Skip fMRI category for now
    if 'fMRI' not in curr_mod_name:
        # Get correlation matrix and p-values


        rho, pval, ax = corrMatrix(curr_data, table=False, plot=False, cmap="RdBu_r",
                                   saveFile = f'{str(corr_out)}/{curr_mod_name}_{group_name}',
                                   fromFile=True)


        # Extract significant correlations
        sign_pairs = [
            (col, i) for col in pval.columns for i in pval[pval[col] < 0.05].index if i != col
        ]
        sign_pairs = list(set(tuple(sorted(pair)) for pair in sign_pairs))  # Remove duplicates

        # Get all combinations
        combos = list(combinations(pval.columns, 2))

        print(f"{len(sign_pairs)} significant correlations out of {len(combos)} feature combinations")

        # Plot some example pairs
        col_ranges = list(range(0, len(sign_pairs), 6))[:2]

        for i in range(len(col_ranges) - 1):
            curr_pairs = sign_pairs[col_ranges[i]:col_ranges[i + 1]]

            # Create subplots with a 1x6 grid
            fig, ax = plt.subplots(1, 6, figsize=(11, 2))
            for pair_n, (x_col, y_col) in enumerate(curr_pairs):
                sns.regplot(
                    x=x_col, y=y_col, ax=ax[pair_n], data=curr_data,
                    scatter_kws={"color": "darkred", "alpha": 0.3, "s": 20},
                    line_kws={"color": "k", "alpha": 0.8, "lw": 2}
                )

            plt.tight_layout()
            plt.show()
            plt.clf()
            plt.close()
        break # remove to plot everything
```

------------------------------ bh_neu features Intercorrelations ------------------------------

```
Loading correlation matrices from disk: D:\NeuroBed_ML\analysis\02 - data_exploration\output\correlations/bh_neu_All
83 significant correlations out of 210 feature combinations
```

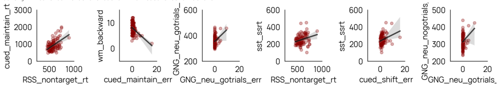

3.2.1 Targets

Here we check the level of significant corrations within feature modalities.

In [23]:
```python
#for group in range(1,5):
curr_data = df_all3[data_cats['targets'].keys()]#.loc[df_reduced['group']==group]
rho,pval,ax = corrMatrix(curr_data,table=True,plot=True)
```

```
                            Disease_unspecific_severity  \
Disease_unspecific_severity                     1.0***
Weight_history_severity                         -0.22*
Eating_unspecific_severity                     0.65***
Eating_specific_severity                       0.77***
Binge_specific_severity                          0.28*

                            Weight_history_severity  \
Disease_unspecific_severity                 -0.22*
Weight_history_severity                     1.0***
Eating_unspecific_severity                   -0.18
Eating_specific_severity                     -0.18
Binge_specific_severity                       0.22

                            Eating_unspecific_severity  \
Disease_unspecific_severity                    0.65***
Weight_history_severity                          -0.18
Eating_unspecific_severity                      1.0***
Eating_specific_severity                       0.71***
Binge_specific_severity                            0.2

                            Eating_specific_severity Binge_specific_severity
Disease_unspecific_severity                  0.77***                   0.28*
Weight_history_severity                        -0.18                    0.22
Eating_unspecific_severity                   0.71***                     0.2
Eating_specific_severity                      1.0***                    0.17
Binge_specific_severity                         0.17                  1.0***
```

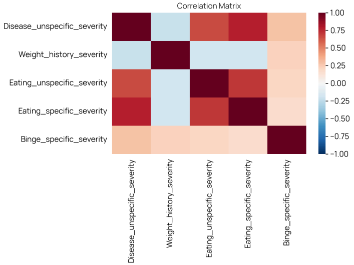

In [24]:
```python
from scipy.stats import f_oneway
from statsmodels.stats.multicomp import pairwise_tukeyhsd
from statannotations.Annotator import Annotator
from statsmodels.formula.api import ols
import statsmodels.api as sm

plotting_df = df_all3.copy()
plotting_df['named_group'] = plotting_df['group'].map(groupNames_rev)

# Prepare long-form dataframe for two-way ANOVA
severity_features = data_cats['targets'].keys()
df_long = plotting_df.melt(id_vars=['named_group'], value_vars=severity_features,
                           var_name='severity_index', value_name='value').dropna()
```

```
# Run two-way ANOVA with interaction
model = ols('value ~ C(named_group) * C(severity_index)', data=df_long).fit()
anova_table = sm.stats.anova_lm(model, typ=2)

print("\n Two-way ANOVA Results (Group x Severity Index):\n")
print(anova_table)

annotate = True # plot test significance

for idx, cols in severity_idx.items():
    if annotate:
        plt.figure(figsize=(4, 6))
    else:
        plt.figure(figsize=(4, 4))
    cols = default_cols(n=2, random=True, n_set=None)
    print(cols)

    feature = idx + '_severity'
    df_plot = plotting_df.dropna(subset=[feature])

    # Violin plot
    sns.violinplot(
        data=df_plot,
        x='named_group',
        y=feature,
        inner='box',
        color='white',
        edgecolor='black'
    )

    # Swarm plot
    ax = sns.swarmplot(
        data=df_plot,
        x='named_group',
        y=feature,
        hue='named_group',
        alpha=0.9,
        palette=2 * cols,
        dodge=False,
        s=4.5,
    )

    # ANOVA
    groups = [group_df[feature].values for name, group_df in df_plot.groupby('named_group')]
    stat, p_anova = f_oneway(*groups)
    plt.title(f"{idx.replace('_', ' ').title()} Severity by Group\nANOVA p={p_anova:.3e}")

    # Post-hoc Tukey HSD if ANOVA is significant
    if p_anova < 0.05:
        tukey = pairwise_tukeyhsd(endog=df_plot[feature], groups=df_plot['named_group'], alpha=0.05)
        results_df = pd.DataFrame(data=tukey._results_table.data[1:], columns=tukey._results_table.data[0])

        # Filter for significant comparisons (p <= 0.05)
        sig_results = results_df[results_df['p-adj'] <= 0.05]

        # If any significant pairs exist, annotate them
        if not sig_results.empty:
            pairs = [(row['group1'], row['group2']) for _, row in sig_results.iterrows()]
            pvals = [row['p-adj'] for _, row in sig_results.iterrows()]

            if annotate:
                annotator = Annotator(ax, pairs, data=df_plot, x='named_group', y=feature)
                annotator.set_pvalues_and_annotate(pvalues=pvals)

    plt.xlabel("Group")
    plt.ylabel(idx)
    plt.legend([], [], frameon=False)  # Remove redundant legend
    plt.tight_layout()
    plt.show()
```

```
 Two-way ANOVA Results (Group x Severity Index):

                                      sum_sq     df          F        PR(>F)
C(named_group)                      2.928026    3.0  31.207749  1.969943e-18
C(severity_index)                   1.797094    4.0  14.365459  4.419042e-11
C(named_group):C(severity_index)    5.338078   12.0  14.223695  1.949071e-25
Residual                           14.886691  476.0        NaN           NaN
['#02577A', '#CBBEB5']
p-value annotation legend:
      ns: 5.00e-02 < p <= 1.00e+00
       *: 1.00e-02 < p <= 5.00e-02
      **: 1.00e-03 < p <= 1.00e-02
     ***: 1.00e-04 < p <= 1.00e-03
    ****: p <= 1.00e-04

BN vs. CON_BED: Custom statistical test, P_val:0.000e+00
BED vs. CON_BED: Custom statistical test, P_val:0.000e+00
BN vs. CON_BN: Custom statistical test, P_val:0.000e+00
BED vs. CON_BN: Custom statistical test, P_val:0.000e+00
```

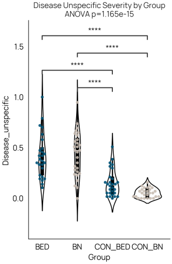


```
['#B4EB93', '#7D7DF6']
p-value annotation legend:
      ns: 5.00e-02 < p <= 1.00e+00
       *: 1.00e-02 < p <= 5.00e-02
      **: 1.00e-03 < p <= 1.00e-02
     ***: 1.00e-04 < p <= 1.00e-03
    ****: p <= 1.00e-04

BED vs. CON_BN: Custom statistical test, P_val:2.360e-02
```

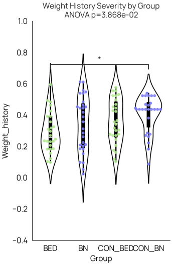


```
['#4747CE', '#EDE93E']
p-value annotation legend:
      ns: 5.00e-02 < p <= 1.00e+00
       *: 1.00e-02 < p <= 5.00e-02
      **: 1.00e-03 < p <= 1.00e-02
     ***: 1.00e-04 < p <= 1.00e-03
    ****: p <= 1.00e-04

BN vs. CON_BED: Custom statistical test, P_val:0.000e+00
CON_BED vs. CON_BN: Custom statistical test, P_val:2.680e-02
BED vs. CON_BED: Custom statistical test, P_val:0.000e+00
BN vs. CON_BN: Custom statistical test, P_val:0.000e+00
BED vs. CON_BN: Custom statistical test, P_val:0.000e+00
```

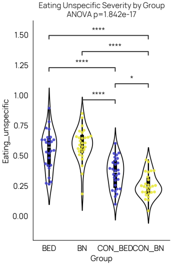


```
['#5597AF', '#A4BADD']
p-value annotation legend:
      ns: 5.00e-02 < p <= 1.00e+00
       *: 1.00e-02 < p <= 5.00e-02
      **: 1.00e-03 < p <= 1.00e-02
     ***: 1.00e-04 < p <= 1.00e-03
    ****: p <= 1.00e-04

BN vs. CON_BED: Custom statistical test, P_val:0.000e+00
BED vs. CON_BED: Custom statistical test, P_val:4.000e-04
BN vs. CON_BN: Custom statistical test, P_val:0.000e+00
BED vs. CON_BN: Custom statistical test, P_val:0.000e+00
```

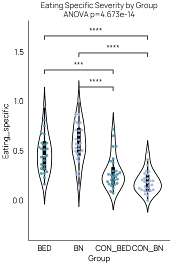


```
['#6A9AB2', '#C79E26']
C:\Users\lenar\AppData\Local\Temp\ipykernel_19504\2265946076.py:46: UserWarning: The palette list has more values (4) than needed (2), which may not be intended.
  ax = sns.swarmplot(
```

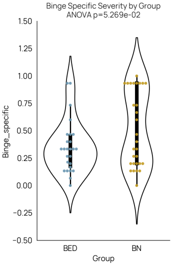

Binge Specific Severity by Group
ANOVA p=5.269e-02
1.50
1.25
1.00
0.75
0.50
0.25
0.00
−0.25
−0.50
Binge_specific
BED
BN
Group

In [ ]:
